# New Advances in Metabolic Syndrome, from Prevention to Treatment: The Role of Diet and Food

**DOI:** 10.3390/nu15030640

**Published:** 2023-01-26

**Authors:** Donatella Ambroselli, Fabrizio Masciulli, Enrico Romano, Giuseppina Catanzaro, Zein Mersini Besharat, Maria Chiara Massari, Elisabetta Ferretti, Silvia Migliaccio, Luana Izzo, Alberto Ritieni, Michela Grosso, Caterina Formichi, Francesco Dotta, Francesco Frigerio, Eleonora Barbiera, Anna Maria Giusti, Cinzia Ingallina, Luisa Mannina

**Affiliations:** 1 Laboratory of Food Chemistry, Department of Chemistry and Technologies of Drugs, Sapienza University of Rome, 00185 Rome, Italy; 2 Department of Experimental Medicine, Sapienza University of Rome, 00161 Rome, Italy; 3 Department of Movement, Human and Health Sciences, Health Sciences Section, University “Foro Italico”, 00135 Rome, Italy; 4 Department of Pharmacy, University of Naples Federico II, 80131 Naples, Italy; 5 UNESCO, Health Education and Sustainable Development, University of Naples Federico II, 80131 Naples, Italy; 6 Department of Molecular Medicine and Medical Biotechnology, University of Naples Federico II, 80131 Naples, Italy; 7 Diabetes Unit, Department of Medicine, Surgery and Neurosciences, University of Siena, 53100 Siena, Italy

**Keywords:** metabolic syndrome, food, food components, diet, metabolomics, biomarkers

## Abstract

The definition of metabolic syndrome (MetS) has undergone several changes over the years due to the difficulty in establishing universal criteria for it. Underlying the disorders related to MetS is almost invariably a pro-inflammatory state related to altered glucose metabolism, which could lead to elevated cardiovascular risk. Indeed, the complications closely related to MetS are cardiovascular diseases (CVDs) and type 2 diabetes (T2D). It has been observed that the predisposition to metabolic syndrome is modulated by complex interactions between human microbiota, genetic factors, and diet. This review provides a summary of the last decade of literature related to three principal aspects of MetS: (i) the syndrome’s definition and classification, pathophysiology, and treatment approaches; (ii) prediction and diagnosis underlying the biomarkers identified by means of advanced methodologies (NMR, LC/GC-MS, and LC, LC-MS); and (iii) the role of foods and food components in prevention and/or treatment of MetS, demonstrating a possible role of specific foods intake in the development of MetS.

## 1. Introduction

Metabolic syndrome (MetS) is characterized by metabolic anomalies including hypertension, central obesity, insulin resistance, and dyslipidemia. The pathogenesis of MetS involves both genetic and acquired factors that play a role in the final pathway of inflammation. In early diagnosis, it is important to change lifestyle and modify risk factors; drug therapy is aimed at treating individual components of MetS. Some nutraceuticals have been shown to have benefits in treatment. This review aims to summarize the epidemiology, pathogenesis, and role of inflammation in MetS. Moreover, this review intends to summarize MetS diagnostic methodologies, the implication of foods and food components, and the new aspects involved in the prevention and treatment of MetS.

## 2. Metabolic Syndrome

### 2.1. Past-Current Definition and Classification

The first definition of MetS was introduced by the Swedish Kylin in 1923 as a syndrome characterized by hypertension, hyperglycemia, and hyperuricemia [1].

Later on, Himsworth divided subjects into insulin-sensitive and insulin-resistant groups, adding important information connected with the pathophysiological background of MetS [2]. During the following years, many different names have been given to the clustering of the components of the MetS, such as syndrome X [3], the insulin-resistance (IR) syndrome [4], and the deadly quartet [5]. However, the first systematic description of the MetS goes back to the late 1980s, when Reaven defined syndrome X as characterized by impaired glucose tolerance (IGT) and hyperinsulinemia associated with high blood triglycerides (TG), high very low-density lipoprotein (VLDL) levels, low high-density lipoprotein (HDL) concentrations, and hypertension [3]. A year later, this definition was integrated by Kaplan by adding the concept of visceral adiposity as another key component of the cluster [6]. Due to the high prevalence, the MetS now represents a dramatic public health concern, and both the medical and scientific communities agree regarding the need to define strategies to stem this emerging pandemic. However, there are yet some controversies related to MetS definition and diagnostic criteria along with some lack of clarity about patients’ identification. In 1999, the World Health Organization (WHO) Diabetes Group centered the definition on the presence of IR, described as IGT or high plasma insulin levels or T2D. To attain a positive diagnosis, another two risk factors need to be present in patients affected by obesity: waist-to-hip ratio (>0.9 or 0.85 for men and women, respectively) and/or (BMI) > 30; hypertension (systolic pressure > 140 mmHg and diastolic pressure > 90 mmHg); dyslipidemia (TG ≥ 1.7 mM and/or HDL < 0.9 mM or <1.0 mM for men and women, respectively); and microalbuminuria, calculated as urinary albumin excretion rate ≥ 20 μg/min or albumin:creatinine ratio ≥ 20 mg/g [7]. In the same year, the European Group for the Study of Insulin Resistance (EGIR) tried to simplify the WHO definition by eliminating the microalbuminuria criterion and giving emphasis to the concept of central obesity rather than overall obesity [8]. Other major criteria have been proposed in 2001 by the National Cholesterol Education Program—Adult Treatment Panel III (ATP III). They excluded the need of demonstrating IR as a mandatory criterion and required the presence of at least three out of five factors to establish the diagnosis [9]. In subsequent years, other associations proposed their definition, and in 2005, the International Diabetes Federation (IDF) tried to unify the definitions of the MetS [1]. Due to the apparent difficulties in establishing clear and universal MetS criteria and the absence of clear evidence for the definition of its exact pathogenesis, the Joint Interim Statement decided to approve a consensus definition by which any patient can be diagnosed with MetS when any three of the following criteria are present [10]:

▪ High waist circumference (WC), whose thresholds depend on populations and country-specific definitions (≥102 cm and ≥88 cm for European men and women respectively) [9];
▪ Blood TG ≥ 150 mg/dL;
▪ Blood HDL cholesterol < 40 mg/dL in men and <50 mg/dL in women;
▪ Blood pressure (BP) ≥ 130/85 mmHg;
▪ Blood fasting glucose ≥ 100 mg/dL.

Due to the high prevalence, MetS now represents a dramatic public health concern, and both the medical and scientific communities agree regarding the need to define strategies to stem this emerging pandemic.

### 2.2. Pathophysiology

As mentioned, MetS consists of a set of conditions that may increase the risk of CVD by promoting the development of atherosclerotic disease but also the risk of chronic nephropathy and T2D [11]. The pathogenetic mechanisms of MetS are complex and have yet to be fully elucidated, but systemic inflammation, also known as chronic low-grade inflammation, has been recognized as an important common factor [12]. A pivotal role is played by adipose tissue, now considered an endocrine organ, undergoing hypertrophy and hyperplasia in response to surplus of caloric intake. An excess of visceral adipose tissue, which is typical of MetS, can therefore lead to a series of metabolic disorders and structural alterations, especially of the vascular and lymphatic microenvironment, which is responsible for potentially lethal hypoxic conditions for the adipocytes further from the vessels. More specifically, hypoxia and lipotoxicity of adipocytes are accompanied by the release of fatty acids and other potential substrates that activate pro-inflammatory pathways within the parenchymal cells of the tissue [13]. This inflammatory process occurs at multiple sites in the visceral adipose tissue, with an enormous, systemic spread of inflammatory cytokines that affect visceral organs and, in particular, insulin-sensitive organs [14]. Ectopic lipid accumulation in muscle and liver has been shown to predispose to IR, which plays a central role in the beginning, progression, and transition of MetS to CVD and T2D [15]. Indeed, IR in adipose tissue impairs the inhibition of insulin-mediated lipolysis, leading to an increase in circulating free fatty acids (FFA) that further inhibits the antilipolytic effect of insulin [16]. In skeletal muscle, these FFA can inhibit the insulin-dependent glucose uptake. On the other hand, IR in skeletal muscle and liver impairs glucose transport and glycogen synthesis, leading to increased insulin secretion by β-cells as a compensatory mechanism to maintain euglycemia and, over time, causing T2D [17]. In addition, a systemic oxidative stress induced by obesity and IR leads to an increased activation of downstream signaling cascades that cause atherogenesis and tissue fibrosis [15]. 

### 2.3. MetS Comorbidities and Complications

The common factor underlying the increased cardiovascular risk in individuals with MetS is apparently the presence of a pro-inflammatory state, which may be associated with an increase in plasma glucose levels [18,19]. T2D indeed often arises on a background of MetS. Diabetes represents a serious health threat as one of the leading risk factors for CVD, end-stage renal disease, and blindness [20]. T2D is frequently preceded by a state of impaired glucose metabolism in which blood glucose or glycosylated hemoglobin (HbA1c) values do not match the criteria for the diagnosis of diabetes but are nevertheless above the normal range. These conditions, collectively referred to as “prediabetes”, do not represent a clinical entity but rather an increased risk for T2D and CVD although such conditions deserve attention and careful monitoring along with lifestyle intervention and correction of other frequently associated risk factors for cardiometabolic diseases [21]. Central obesity has long been considered as a prerequisite of MetS. Obesity per se is a risk factor for metabolic diseases including dyslipidemia, T2D, hypertension, and non-alcoholic fatty liver disease (NAFLD) and is related to several complications, i.e., obstructive sleep apnea (OSA) [22], which contributes to poor quality of life and adverse cardiovascular outcomes [23]; joint disorders such as osteoarthritis [24]; and an increased incidence of cancer [25] and cancer-related mortality, most likely attributable to chronic hyperinsulinemia [26]. However, adipose tissue distribution is of great relevance for obesity-related comorbidities. Indeed, visceral obesity, which is characterized by preferential accumulation of fat in intra-abdominal, epicardial, and perivascular depots as well as ectopic fat accumulation in liver, skeletal muscle, and pancreas, is associated with a peculiar metabolic profile, including IR, local and systemic inflammation, and secretion of pro-inflammatory and pro-thrombotic cytokines and adipokines. Interestingly, these metabolic abnormalities, including an increased risk of CVD, arise even in lean individuals who accumulate fat intra-abdominally (the so-called “normal-weight obesity”). On the contrary, subjects who preferentially accumulate fat subcutaneously in the lower body seem to be metabolically healthy and are less prone to develop metabolic comorbidities [27,28]. Therefore, it has been proposed to consider other measures besides BMI in the assessment of patients with obesity, such as WC, which highly correlates with intra-abdominal fat content, to better define the phenotype and assess individual metabolic and cardiovascular risk. 

Another critical risk factor for CVD is an elevated blood pressure, a common feature of MetS [29] occuring five times more frequently in subjects with visceral obesity than in normal-body-weight subjects [30]. Systemic arterial hypertension is the most common and preventable cause of CVD and represents the leading cause of premature death worldwide [31]. Dyslipidemia is also responsible for significant morbidity and mortality, and international guidelines have focused on the need of intensive LDL-C lowering therapy to reduce CVD risk [32,33]. In addition to the above-mentioned comorbidities, MetS may be complicated by other disorders increasing disease burden like NAFLD, which is considered as the hepatic manifestation of MetS by some authors [34]; major depressive disorder (MDD) [35], which shares the pheripheral inflammation component with MetS [35]; male infertility, possibly by means of a detrimental effect by oxidative stress on sperm quality [36]; polycystic ovary syndrome (PCOS) [37]; different degrees of cognitive impairment and earlier progression to dementiae (i.e., Alzheimer’s disease and vascular dementia) [38,39,40,41], especially among MetS subjects with T2D or prediabetes; and sarcopenia [42]. Finally, with the spread of SARS-CoV-2 infection worldwide, it has become evident that pre-existing metabolic disorders are risk factors for more severe forms of COVID-19 and COVID-19-related mortality but also likely for other communicable infectious diseases. Specifically, it is likely that MetS-induced chronic inflammation predisposes to COVID-19 cytokine storm, influencing patient clinical outcomes [43]. Indeed, patients with obesity are more prone to develop infectious diseases and more severe clinical complications; the relationship between obesity and infections has not been fully elucidated, but it is assumed to stem from an alteration in both innate and adaptive immune responses, further aggravated by several obesity-related co-factors (e.g., vitamin D deficiency, respiratory impairment, and skin and subcutaneous tissue changes) [44]. The global prevalence of severe forms of COVID-19 among MetS patients requires prophylactic strategies to reduce the risk of infection in these high-risk individuals as well as timely and crucial treatment and monitoring the post-infection course [45]. Nevertheless, since MetS patients are more susceptible to severe clinical manifestations of infectious diseases, future characterization of precocious biomarkers will aid in identifying patients at higher risk of complications.

## 3. Methodologies for Timely Prediction and Diagnosis of MetS

As mentioned earlier, subjects affected by MetS are characterized by different metabolic derangements which may be subclinical at the time of presentation (e.g., impaired fasting glucose) but have the potential to progress to individual clinical entities (e.g., T2D). As a consequence correctly diagnosing MetS requires continuous monitoring, since clinical inertia can unfavorably affect both patient compliance to medical therapy and early identification of MetS comorbidities. Furthermore, through time different diagnostic criteria for MetS have been proposed by medical scientific societies: this ambiguity poses the need for new, unbiased methods based on molecular features.

**Figure 1:**
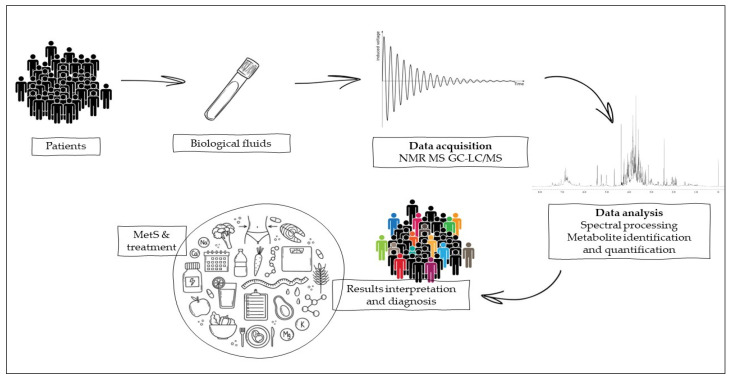
Metabolomics experimental work from biological samples of patients for the interpretation of the results obtained by nuclear magnetic resonance (NMR) or mass spectroscopy (MS), coupled to targeted techniques such as gas and liquid chromatography (GC and LC).

In this context, the metabolome plays a key role, as this set of metabolites is able to vary in a particular metabolic condition such as MetS (hypertension, central obesity, IR, and dyslipidemia), which is dependent on both genetic and acquired factors. The small metabolites generated by this multifaceted metabolic disorder all contribute to the modulation of the metabolome and can be assessed by metabolomics, which in turn allows the observation of quantitative changes in molecules suggestive of MetS comorbidities. This bioanalytical technique represents a challenge for diagnosing a pathological state and evaluating the prognosis of nutritional therapy on patients suffering from MetS [46] -as showed in Figure 1. The study of the metabolome could be conducted by both untargeted analyses (e.g., nuclear magnetic resonance, NMR, and mass spectroscopy, MS) and targeted analyses such as chromatographic techniques coupled to mass spectrometric detection (HPLC, UHPLC, GC, and supercritical fluid chromatographic). 

Combined metabolome analysis of biological fluids and clinical markers represents a promising approach for metabolic monitoring MetS patients. Selected works concerning predictive and diagnostic MetS methodologies are listed in Table 1.

### 3.1. Nuclear Magnetic Resonance 

In the diagnosis of MetS, the NMR approach should provide a good alternative to detect an earlier stage of this condition compared to the traditional methods; the latter are useful when the disease status is well-established. 

The biological fluids most commonly used in NMR metabolomic studies are plasma, serum, urine, and feces [65]. For human studies, plasma and serum are preferred because they are easy to collect, and their metabolome reflects changes in metabolism at an individual level. Novel promising biomarkers could be metabolites such as glucose, lactate, uric acid, citric acid, *p*-cresol sulfate, imidazole, histidine, branched-chain amino acids (BCAA), aromatic amino acids (AAA), glutamate and glutamine, propionyl carnitine, lipids, and many others. Glucose is a carbohydrate used as biomarker to diagnose a change in carbohydrate metabolism (glucose homeostasis) involved in IR or T2D, which are part of the multifactorial nature of MetS. In a recent study, NMR analysis of the urine profile revealed how patients affected by T2D show metabolome variation in comparison with healthy subjects. This change consists of a decrease in the concentration of creatinine, *N*-acetyl groups (glycoproteins), allantoin, glutamate and glutamine, and histidine with an increase in glucose [47]. A decrease was observed in other metabolites such as valine, leucine, and isoleucine (BCAA) as well as *N*-butyrate, citrate, and lactate, with the last metabolite being involved in glucose metabolic pathways [48]. Thus, lactate represents an eligible marker to diagnose the pathologies characterized by carbohydrate metabolism disruption at the early state of disease. In several NMR studies on biofluids, increased lactate has been observed in both the urine and blood of patients with T2D and especially in overweight subjects [49]. In a metabolomic study recruiting 63 patients with MetS, 82 MetS and asymptomatic hyperuricemia (HUA) patients without clinical gout (uric acid > 240 μmol/L), and 61 healthy control subjects, the serum samples were analyzed using H-NMR spectroscopy, displaying a tendency for metabolic disorders to grow in patients with both MetS along with an increase in serum uric acid. The results showed a significant increase in lipid, TG, and urine glucose levels and remarkable lowered levels of glutamine, trimethylamine (TMA), isoleucine, alanine, lysine, 3-hydroxybutyrate, glutamate, citrate, proline, glycine, tyrosine, and 1-methylhistidine [50], supporting uric acid as an upstream inducer in the pathogenesis of hypertension, IR, diabetes, dyslipidemia, and obesity [66]. Therefore, it is a useful marker in the diagnosis of the early state of MetS, and it was proposed as one of its diagnostic criteria [67]. The disruption in fatty acid metabolism that usually occurs in MetS causes an increase of acylcarnitine. Therefore, acylcarnitine could be an NMR-predictive biomarker of T2D and other pathological pathways involved in the framework of MetS [51]. Indeed, the metabolomic analysis conducted on human blood or serum samples allows the qualification and quantification of several acylcarnitine, particularly propionylcarnitine (C3), which is paramount role in interrupted lipid and amino acid metabolism in patients suffering from MetS [68]. Since essential amino acids can only be introduced by diet, and their blood levels are regulated by catabolic processes, their plasma levels correlate with several pathologies, including the metabolic ones. Many studies conducted on both animal and human models correlated BCAAs and AAAs (including phenylalanine and tyrosine) with MetS and all the single diagnostic features [52]. Several metabolomic NMR studies on mice or humans showed an increase of BCAAs in subjects affected by disordered lipid and carbohydrate metabolism [53] such as IR and obesity [54]. NMR analysis would best predict MetS by using BCAA and AAA as two different clusters, both of which precede hyperglycemia [55]. Other amino acids, such as glutamine and glutamate, are also useful biomarkers of metabolic alterations typical of MetS. An NMR study on animal models highlighted how glutamine increases in obese mice fed a high-fat diet (HFD) [56]. Won et al. [57] showed, based on NMR analysis of serum and urine of murine model, how an HFD could lead a modification in metabolomic profile in which products of gut microbiota metabolism (choline, *p*-cresol, 3-hydroxybutyrate, hippurate, and trigonelline) and products of glucose metabolism such as citrate are significantly increased, showing a variability in metabolomic profile based on IR and dyslipidemia typical of MetS. MetS includes a low degree of systemic inflammation, and metabolomic analysis using NMR has improved the diagnosis of a systemic inflammatory state despite of traditional clinical methods based on immunochemical tests such as ELISA. The glycosylation products such as GlycA [69] could be NMR biomarkers to predict an inflammatory state typical of metabolic dysfunction [70]. A study on plasma metabolic profile was performed by NMR in a pediatric sample, displaying an increase in *N*-acetyl glycoprotein among patients with obesity compared with healthy controls [71]. One more important marker to diagnose MetS is represented by lipid profile. An NMR study of serum metabolic profile in overweight women with or without MetS exhibited a variation in circulating lipid composition, with some polyunsaturated fatty acids (PUFAs) being characteristic of MetS [58]. However, lipidomic analysis conducted by NMR on whole-blood samples presents several difficulties, so it would be better to use additional chromatographic techniques [72,73]. Metabolomics measurements on biofluids of several cohorts of patients allow for the assessment of both the prediction of disease processes implicated in MetS and the progression of the disease condition during drug treatment, eventually bringing NMR metabolomics into the clinical practice [74].

### 3.2. MS-Chromatographic Techniques 

As mentioned in the previous section, lipidomic analysis conducted by NMR needs to be supported by chromatographic techniques associated with MS, which allowed lipidomic to make great strides. The metabolomic analyses performed using techniques such as HPLC-TOF-MS, UPLC-QTOF-MS, HPLC-MS, GC-MS, and LC-MS revealed that several metabolites were dysregulated in the plasma of patients with MetS, especially some lipid species such as FFA, phosphatidylcholines, ether phosphatidylcholines, and others [59], proving useful in the clinical practice to diagnose metabolic changes. From a lipidomic study conducted by GC-SIM-MS on human serum samples, an identical FFA profile was observed in both healthy subjects and patients affected by MetS. A substantial increase in lipids concentration with a direct correlation between serum FFA and increased in HDL, TG, and FBG (fasting blood glucose) levels, all factors observed to be included in MetS [60]. Several studies on human plasma samples have shown that sphingolipids are good biomarkers of metabolic abnormalities; for example, from LC-MS/MS analysis in normoglycemic patients with obesity and MetS, a sphingophospholipidome change is observed before the onset of hyperglycemia [75]. In another cohort study of a Chinese population performed by HPLC-MS/MS, two subclasses of sphingolipids were significantly associated with MetS: some ceramides (C18:0, C20:0, C24:1, C22:1, C20:1) and hydroxyl sphingomyelin. Ceramides were associated with a higher risk of MetS because they result in disruption of insulin action [61], while high levels of hydroxyl sphingomyelin were associated with a lower risk of MetS becausethey prevent dyslipidemia by reducing intestinal absorption of cholesterol, TG, and fatty acids [62]. In addition to lipidomics, metabolomics analysis performed by chromatographic techniques combined with MS was used to find other potential biomarkers that could diagnose MetS dysfunction. For example, Lin et al. identified several metabolites such as 2-hydroxybutyric acid, inositol, and D-glucose that are overreoresented in the serum of patients with MetS compared with healthy subjects [63] due to the presence of IR and compromised glucose regulation. Another cohort study by LC-HRMS on urine samples was able to show increased tryptophan metabolites in MetS subjects compared with healthy controls. The metabolites identified were indole-3-acetic acid, indole-3-acetic acid-O-glucuronide, *N*-(indol-3-ylacetyl) glutamine, indole-3-carbaldehyde, hydroxyhexanoycarnitine, and indole-3-carboxylic acid. These indole compounds are related to the pro-oxidative and altered conditions in fatty acid oxidation that accompany MetS [64]. Thus, all these metabolites can be used as biological markers in patients with obesity and diabetes to evaluate the MetS state.

### 3.3. Metabolic Syndrome Management, Interventions, and Challenges

Since MetS includes a cluster of CVD risk factors, the primary aim of the clinical management is to minimize the occurence of major CV events (stroke, infarction) through a combined action on these factors. The initial goal is acting on modifiable baseline conditions such as obesity, physical inactivity, and atherogenic diet through lifestyle changes [19]. Lifestyle recommendations include smoking cessation, physical activity (30–60 min per day), the Mediterranean diet with or without energy restriction (specific dietary recommendations include limited intake of saturated and trans fats, sugar-sweetened beverages, alcohol, and salt), and, for overweight and patients with obesity, a healthy specialist-designed diet to achieve the desirable BMI (<25 kg/m^2^) [76]. Due to its high morbidity rate, MetS is a relevant health problem that to date remains unresolved at a global level, with a 30% of the population being affected [77,78]. Moreover, MetS has been associated with poor health-related physical and mental quality of life, suggesting that a multidisciplinary approach outlook needs to be implemented. In particular, it might be reasonable to consider its cluster of metabolic abnormalities as an “alarm bell” that does require immediate intervention. Thus, therapeutical approach to MetS requires lifestyle correction, both nutritional and physical activity protocol, and often pharmacological therapy. Physical activity has proven helpful in decreasing body weight, improving peripheral IR, but also in reducing anxiety [79,80]; in patients with MetS [81,82], however, few studies have been performed evaluating the impact of a physical activity program on anxiety and depression [83,84,85]. Even though patients with MetS are highly motivated to improve their health, they are surely in need of both support and advice on how to be physically active. A recent study demonstrated how personalized consultation and modifications resulted in increased physical activity [86]. Each patient with MetS necessitates monitoring and follow-up sessions to ensure the adherence and the successful completion of the training program to achieve initial results and improve their health [87]. The priority intervention is represented by blood LDL-C reduction, whose clinical target depends on the patient’s estimated absolute cardiovascular risk [88,89]. Other strategies focus on pharmacological treatment of high blood pressure (using angiotensin-converting-enzyme (ACE) inhibitors as first-line therapy) [90], elevated fasting blood glucose and IR (using metformin as first-line therapy) [91], and pro-thrombotic status (primary prevention with low-dose aspirin or other antiplatelet agents when needed) [92]. Of note, since these patients may be affected by MetS for the rest of their lives, adopting and maintaining a new lifestyle may prove difficult; thus, their re-education both in terms of their dietary patterns and physical activity emerges as a potential solution. These observations have generated much interest in the scientific community on the effectiveness of prevention programs based on lifestyle and dietary pattern modifications.

## 4. New Aspects Implicated in the Prevention and Treatment of MetS

### 4.1. Gender Medicine and Metabolomics

Lately, a consistent number of studies has been focusing on how to tailor medical nutrition therapy for individual patients within MetS populations. A study from Christesen et al. [93] investigated gender-specific clinical outcomes in overweight, pre-diabetic patients following an 8-week fixed, low-energy diet (LED). A MetS score was computed from a new equation including all of the five variables identifying MetS according to Alberti et al. [8], from which a standardized z-score was calculated. Men showed larger reductions in MetS z-score and body weight, *C*-peptide levels, and fat mass (FM) than women even after adjusting for differences in % weight loss. On the other hand, women displayed an undesirable reduction in HDL-C, fat-free mass (FFM), and bone mineral content (BMC) [93]. On the metabolomics side, Geidenstam et al. found that baseline xylitol levels in serum and decreased levels of methyladenosine, alanine, proline, trans-cinnamic acid, tyrosine, and BCAA in serum in response to a 1-year non-surgical weight loss program were a predictor for achieving ≥10% weight loss [94]. In another study, the same authors created a metabolic risk score based on 42 metabolites associated with a BMI change in order to predict future weight gain [95]. Particularly, the increase of 35 metabolites levels was associated with smaller weight gain, whereas the other seven metabolites were associated with a larger weight gain. Finally, eight metabolites (specifically triacylglycerol 56:6 and 56:2, malate, niacinamide, sphingomyelin 24:0, uridine, tyrosine, and xanthine) were identified as a predictor of the risk of future weight gain. Model’s variance was not fully explained by anthropometric, lifestyle, and glycemic predictors; at the same time, this score showed a strong positive correlation with insulin sensitivity markers and a negative correlation with T2D risk [95]. 

The PREVENTOMICS study is developing a personalized nutrition algorithm based on five different metabotypes taking account of (1) carbohydrate metabolism, (2) lipid metabolism, (3) oxidative stress, (4) inflammation, and (5) microbiota composition. At present, the study is still ongoing [96]. 

The Food4Me study enrolled patients into four different dietary advice groups: one based on guidelines for the general healthy population (non-personalized group), one based on dietary intake data alone, one based on dietary intake data and phenotypic data, and one based on dietary intake, phenotypic, and genotypic data combined. No clinically significant difference in terms of body weight or WC change was observed between the three personalized groups and the control group; however, the personalized groups achieved better eating patterns and healthier diets [97]. A sub-study of this project identified three metabotypes through statistical cluster analysis of 27 biomarkers (including cholesterol, fatty acids and carotenoids); each metabotype was assigned a specific dietary counselling treatment congruent with the patient’s metabolic features and nutritional requirements. Outcomes comparable with those achieved by traditional counseling from nutrition experts were observed [97]. Another application of metabotypes is glucose homeostasis. The PREDICT twin study [98] tested the ability of a multi-omics machine learning model on predicting post-prandial glucose, TG, and insulin serum levels after food consumption, with promising results (r = 0.47 for TG; r = 0.77 for glucose). In addition, authors investigated the potential benefit of using an electronic app to provide patients with personalized nutritional counseling on portion sizes and food items’ frequency of consumption [98]. Another recent study, the PERSonalized glucose Optimization through Nutritional intervention (PERSON) study, focused on insulin sensitivity among patients with a BMI 25–40 kg/m^2^ and either liver insulin resistance (LIR) or muscle insulin resistance (MIR). Participants within each group were subsequently randomized into isocaloric isoglucidic diet (moderate-fat diet rich in monounsaturated fatty acids, deemed optimal for MIR patients, or a low-fat, high-protein diet rich in fiber, deemed optimal for LIR patients) [99]. The primary outcome was the change in oral disposition index between groups. Comprehensive analysis of collected data is currently lacking, but this study could help elucidate metabolic markers of obesity-induced insulin resistance. Qiong Wu et al. [100] implemented a metabolite risk score (MRS) based on five metabolites (docosapentaenoic acid or DPA, lysophosphatidylcholine or LysoPC (14:0), LysoPC (15:0), propionyl carnitine, and L-phenylalanine) that displayed a dose–response relationship with MetS and correlated metabolic abnormalities. The authors identified two SNPs (rs1952458 and rs11635491) that were marginally correlated with MetS; an association between higher LysoPC (15:0) levels and the risk of overfatness, dyslipidaemia, hyperuricemia, and IR was apparent [100].

Finally, categorizing patients according to their metabolic phenotype (based on cardiometabolic biomarkers, plasma lipoprotein and fatty acid profiles, and postprandial levels of insulin or glucose) could contribute to predicting clinical response to nutrition therapy [101].

### 4.2. Dietary Patterns

Numerous studies have shown that good human health depends largely on diet, which helps build, regenerate, and maintain the body and provide the energy essential for the body to function properly. Increasing consumption of some foods and avoiding or reducing that of others can help maintain a state of well-being. In the last decades, the prevalence of MetS has dramatically increased, likely due to changes in lifestyle factors, socioeconomic status, and dietary habits. One of the biggest challenges in the management of MetS is to mitigate the risk associated with the MetS components though lifestyle intervention, as mentioned elsewhere in this review. Observational studies reported potential associations between different dietary patterns and the risk of MetS [102]. In particular, the results from cross-sectional studies showed that a healthy dietary pattern is associated with a lower prevalence of MetS, whereas a Western/unhealthy diet (characterized by high intakes of red and processed meat, refined grains, alcohol, and fried foods) is associated with an increased risk for MetS [102].

Several studies have been specifically conducted on the Mediterranean diet (MedDiet): for instance, the PREDIMED study showed a 40% reduction in T2D incidence in the intervention group (MedDiet + extra-virgin olive oil) compared with a low-fat diet (LFD). Moreover, a recent meta-analysis of 50 prospective and randomized controlled studies established a positive association between adherence to a MedDiet pattern and a 50% reduction in MetS incidence [103]. The modulation of epigenomics behind metabolic homeostasis and dysfunction has also been taken into consideration [104]. Adherence to a short- or long-term MedDiet impacts miRNA expression profiles and transcription of genes involved in inflammatory, adipogenic, and atherogenic pathways of MetS pathogenesis [104]. From the same line of evidence, Marques-Rocha JL et al. revealed an altered expression of miRNAs involved in the pathogenesis of CVDs in patients with MetS following an 8-week hypocaloric MedDiet [105]. As part of a MedDiet pattern, regular consumption of ω-3 PUFAs increases fatty acid oxidation in the liver and adipose tissue and regulates the peroxisome proliferator: indeed, EPA-rich oil (EO) activates PPAR α and PPAR β/γ receptors, with minimal effects on PPARγ, liver X receptor, retinoid X receptor, farnesoid X receptor, and retinoid acid receptor γ (RAR γ) [106] and reduces lipogenesis, decreasing the expression of sterol response element binding protein-1c [107]. However, *n*-3 PUFAs could also influence miRNAs activity on target genes, affecting lipid metabolism, adipokine levels, adipose tissue flogosis, and adipogenesis at the epigenetic level [104,108]. Castro-Barquero et al. [109] compared different dietary approaches (DASH diet, MedDiet, Nordic diet, intermittent fasting, ketogenic diets, low-fat diet, high-protein diet, and plant-based diet), focusing on their effects on individual MetS components. All diets showed a positive and specific impact on MetS; for instance, intermittent fasting seemed to improve weight loss, IR, dyslipidemia and hypertension, thus decreasing T2D and CVD risk [110,111,112]. However, a major issue is its feasibility since it requires specialized staff and the patient’s compliance to a restricted time frame for eating. Authors agreed that, for preventing and managing MetS, MedDiet should represent the first line of treatment; furthermore, global dietary pattern changes should be preferred to dietary restriction of single macronutrients or food groups [109].

## 5. Food and Foods Components

In the previous chapter the relationship between dietary patterns and components of MetS was evaluated. The results indicated that a diet based on healthy food choices, i.e., the consumption of a diet rich in cereals, fish, fruit and vegetables is associated with a healthier metabolic profile and lower risk of MetS.

**Figure 2:**
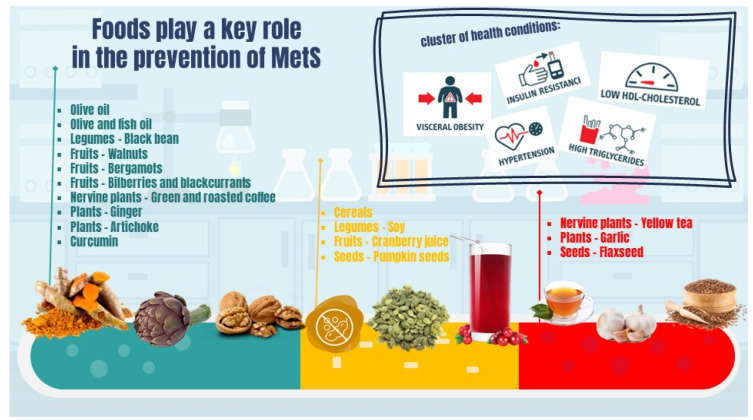
The role of foods and food components in MetS.

Growing evidence supports the role of foods and individual nutrients (and non-nutrients) in preventing and managing MetS manifestations and comorbidities. The role of various foods and food components in the prevention or treatment of MetS is reported in the following section and summarized in Figure 2 and Table S1. Inclusion and exclusion criteria used for the selection of the reported studies are described in supplementary material.

### 5.1. Seeds

The use of plant seeds to treat disorders of the MetS is becoming increasingly important. In this serction, we will specifically analyse two types of plants: pumpkin (*Curcubita pepo* L.) and flax (*Linum usitatissimum* L.). 

In pumpkin seeds, high amounts of protein (14.3–38%) and fat (21.9–54.9%) are present. Unlike fruits seeds, they are also a good source of amino acids (17 of the 20 amino acids that make up human protein) [113]. 

Chenni et al. [114] conducted an in vivo study to investigate the potential beneficial effects of pumpkin seeds on MetS risk factors such as IR, glucose tolerance, oxidative stress, and the inflammation status in rats with high-fructose-induced MetS. Twenty-four male Wistar rats were divided into four groups and fed isoenergetic diets supplemented for eight weeks: C group (20% casein), P group (20% casein and purified pumpkin seed proteins by oral at the dose of 1 g/kg/day), C-HF group (20% casein enriched with 64% fructose), and P-HF group (20% casein and purified pumpkin seed proteins, administered by oral gavage at the dose of 1 g/kg/day and enriched with 64% fructose). Several parameters were evaluated during the study, such as body weight, food intake, weight gain, food efficiency, BMI, and adiposity index. Wet weights of muscle tissue, white adipose tissue (WAT), and epididymal adipose tissue (EAT) were then evaluated. At the end of the 60-day period, even if food intake of the C-HF group was lower than in the C group, probably due to high levels of energy production, the wet weight of the WAT was higher as compared to the C diet. It was seen that pumpkin seed intake reduced adiposity index when compared to the C-HF group. Plasma glucose and insulin values were assessed. Insulinogenic index (IGI) is the ratio between insulin and glucose concentration. Increased WAT weight due to the HF diet may be responsible for alterations in the HOMO-β index (indicating pancreatic beta cell function), insulinogenic index, insulin sensitivity, D-glucose, and plasma insulin concentrations. All these effects may be due to an alteration in the glucose recognition process by beta cells or defects in glucose transporters. Plasma D-glucose concentration and area under the curve (AUC) were higher in fructose feeding than in the control group. Use of pumpkin seeds in the P-HF group decreased plasma glucose and AUC by 29% compared to the C-HF group. This alteration appears to be due to a change in insulin sensitivity, as indicated by the increase in HOMA-IR, HOMA-β, and insulinogenic index. The improvement in adiposity without weight loss suggests that pumpkin seed proteins fight the development of glucose tolerance and IR induced by chronic fructose intake. The decreased IR may result from enhanced peripheral utilization of glucose and/or increased insulin receptor binding. It is also possible that this fructose-induced IR may be counteracted by the high amounts of arginine and cysteine in pumpkin seeds. 

It was also evaluated whether pumpkin seeds had antioxidant activity in fructose-induced MetS rats. Indeed, chronic administration of fructose led to high concentrations of thiobarbituric acid-reactive substances of (TBARS) and lipid tissue hydroperoxide (LPH) in WAT and muscle tissue, with changes in the antioxidant enzymatic systems such as GSH-Px, SOD, and CAT activities. It was also seen that the fructose-rich diet led to a decrease in nitric oxide (NO) levels, a finding already observed in patients with MetS. Pumpkin seed decreased both TBARS and LPH levels in the WAT and increased CAT enzymes. Finally, anti-inflammatory activity has also been observed in pumpkin seeds. TNF-α and IL-6 levels were measured in the four groups, and the values were higher in the C-HF and P-HF groups than in the P and C groups. This increase could be due to NF-κB activation, which in turn increases the expression of pro-inflammatory cytokines in a fructose-rich diet. This could be due to the IR that develops in individuals with MetS (the anti-inflammatory effect of insulin is lost). Adiposity can also cause inflammation, and a high-fructose diet leads to high free fatty acid quantities, resulting in inflammation. TNF- α (but not IL-6) levels decreased following the intake of pumpkin seeds. Thus, this anti-inflammatory effect may be due to the decrease in IR and adiposity levels in a pumpkin-seed-rich diet. This study therefore shed light on the properties of pumpkin seed proteins, showing their potential in the treatment and prevention of MetS.

Regarding flaxseed, Atefeh et al. [115] evaluated the effects of flaxseed oil, compared to sunflower oil, against MetS. In this randomized controlled clinical trial, authors analyzed the effects of the two oils on coagulation score, totalTAC, and inflammatory parameters (IL-6) in 60 patients with MetS (aged between 30 and 60 years). They divided the patients into two groups: group one was given 25 mL/day of flaxseed oil and group two 25 mL/day of sunflower oil for eight weeks. Flaxseed oil is a good source of alpha-linolenic acid (ALA). Further important compounds present in flaxseed oil are lignans and fiber. These products also contains vitamin E in the form of γ-tocopherol and thus protects cellular membrane lipids from oxidation. At the beginning and at the end of the study, 2 mL venous blood samples were taken after a 12-h overnight fasting. Serum TAC and IL-6 level was measured using an ELISA kit. The coagulation score was calculated by centrifuging plasma. At the end of the study, a decrease in IL-6 production was observed, resulting in a reduction of the inflammatory state in patients with MetS. ω-6 and ω-3 fatty acids induced changes in both cyclooxygenase and lipoxygenase products, such as reduction in production of prostaglandin E2 and leukotriene B4. The reduction of these two eicosanoids could explain the decrease in IL-6. However, replacement of various fatty acids in the diet can have different effects. As noted in other studies, the low and high doses of flaxseed oil were unable to change IL-6 and some other inflammatory parameters in patients with MetS [116]. These different results may come from the baseline levels of inflammatory markers as well as habitual diets, which are the main influential factors. As regards TAC and coagulation score, no significant differences between the two groups were found. One work has shown that *n*-3 fatty acid improved endothelial function and platelet aggregation markers in patients with coronary heart disease as well as in healthy cigarette smokers [117]. It seems that inflammatory status affects endothelial dysfunction in patients with MetS and eventually increases the coagulation factors. However, details and mechanisms of the antithrombotic effects of ALA are unclear. Therefore, further studies on the issue are needed.

### 5.2. Plants

Over the years, several plants have been studied to evaluate their beneficial effects on MetS. A double-blind, randomized controlled clinical trial was conducted in order to investigate the effect of garlic (*Allium sativum* L.) supplementation on MetS manifestations including insulin resistance, fatty liver index (FLI), and orexigenic stimuli [118]. Ninety participants were divided into two groups; the first group was given four garlic powder tablets per day (400 mg of garlic powder per tablet), while the second group received a placebo (also four times a day). The test lasted three months, and patients were asked to take two tablets one hour before lunch and another two one hour before dinner (due to reduction of garlic bioavailability after protein intake). During the study, various parameters such as DBP and SBP, IR, BMI, fatty liver, serum insulin index, and even appetite status were evaluated. The latter was evaluated using questions such as “how strong is your feeling of hunger, fullness”, and “how much food can you eat right now”, with answers of “not (much) at all (score 0)” to “extremely/an extreme amount (score +100)”, as well as a question assessing “how strong is your feeling of desire to eat”, with answers of “extremely/an extreme amount (score 0)” to “not (much) at all (score +100)”. The questionnaires were answered 5 min before lunch. In the group given garlic tablets, there was an improvement in the MetS manifestations, including DBP, SBP, WC, TG, and HDL-C. Furthermore, a marked decrease in γ-glutamyltransferase (GGT), FLI, and HOMA-IR was observed. Finally, the garlic-treated group showed an increase in fullness as well as a decrease in hunger, desire to eat, and ability to eat.

Another interesting study was conducted by Rezazadeh et al. [119], where the effects of artichoke (*Cynara scolymus*) leaf extracts (ALE) were evaluated against MetS by assessing their antioxidant activity. This double-blind placebo-controlled randomized clinical trial was conducted on 80 patients with MetS (aged between 20 and 25 years). The subjects were divided into two groups: the ALE group that received 1800 mg/day of artichoke leaf extracts distributed in four tablets per day and the placebo group that received tablets containing cornstarch, lactose, and avicel, all for a duration of 12 weeks. The patients were instructed to continue their usual food intake and physical activities over the study period. Several parameters such as the concentration of HDL-C, TG, and FBS in serum were measured before and after the study to assess whether and how ALE supplementation could have beneficial effects against MetS. Antioxidant activity was assessed by monitoring the levels of glutathioneperoxidase (GPx) and superoxide dismutase (SOD) of red blood cells (RBC) and serum total TAC. At the end of the study, it was seen that ALA supplementation decreased ox-LDL and TG levels in patients with MetS, while there were no statistically significant differences between the two groups with regard to SOD, GPx, and TAC. The observed antioxidant effect of ALE on ox-LDL reduction is probably due to some of its constituents, such as flavonoids and hydroxycinnamic acids, acting as donors of hydrogen ions and chelate metal ions.

Another plant that is very important due to its innumerable beneficial properties on human health is ginger (*Zingiber officinale* Roscoe). Ginger has multiple biological activities, including antioxidant, anti-inflammatory, antimicrobial, cardiovascular protective, antiobesity, antidiabetic, etc. The bioactive compounds mostly found in ginger are phenolic (gingerols, shogaols, and paradols) and terpene (β-bisabolene, α-curcumene, zingiberene, α-farnesene, and β-sesquiphellandrene) compounds. Polysaccharides, lipids, organic acids, and raw fibers are also present in ginger. 

Li Y. et al. [120], conducted an in vivo and in vitro study to evaluate the effect of ginger extracts against IR, one of the key factors in MetS. The in vivo experiment was performed in rats fed a diet rich in fat and carbohydrates (HFHC). They divided the animals into five groups, with seven rats in each group: in particular, group 1 was given a standard diet (control group), group 2 was HFHC control, groups 3 and 4 were given ginger extracts (100 mg/kg and 200 mg/kg per day), and group 5 was treated with metformin (200 mg/kg). After 10 weeks of treatment, a marked reduction in blood glucose was observed in group 4 treated with 200 mg/kg per day of ginger extract compared to the HFHC control group. The blood insulin concentration in the HFHC group was much higher than in group 1, and treatment with ginger extract (200 mg/kg per day) resulted in a decrease in serum insulin level in HFHC-diet-fed rats and an improvement in insulin sensitivity, the same positive effects as in group 5 treated with metformin. In regards to the in vitro test, it was found that (S)-[6]-gingerol, a major active component in ginger, increased activated protein kinase a AMPKa phosphorylation in L6 skeletal muscles cells and increased mRNA expression of PGC-1a within 5 h, a very important activity since down-regulation of PGC-1a and defects in mitochondrial function are closely related to the pathogenesis of IR and T2D. These results show that ginger has beneficial effects, likely modulating glucose metabolism in rats fed a high-calorie diet, suggesting that ginger might be effective in preventing the development of MetS and T2D.

### 5.3. Nervine Plants

In this section, the so-called nervine plants are reported not for their stimulating effects but for their polyphenolic fraction, which has shown remarkable activities in preventing and treating MetS. We will focus on coffee and other similar plants.

A randomized, controlled, crossover clinical study was carried out by Sarria et al. [121] to evaluate the effects of green and roasted coffee against MetS. The 52 participants (men and women aged between 18 and 45 years) were divided into two groups: normocholesterolaemic (total cholesterol (totChol) < 200 mg/dL) and hypercholesterolaemic (totChol > 200–240 mg/dL). After this, subjects were randomly distributed in two groups so that half the participants firstly consumed the green/roasted coffee blend, and the other half had the control drink (water or an isotonic caffeine- and polyphenol-free drink) for 8 weeks. Then, after a three-week washout stage, subjects changed to drink the other beverage during the same time (8 weeks). The doses of the coffee blend were 2 g three times a day (the control drink was also taken three times a day). The soluble coffee product used was a commercial green/roasted mixture (35:65, *w*/*w*). Total soluble polyphenols and methylxanthine were analyzed by high-performance liquid chromatography with diode array detection (HPLC–DAD); coffee contained 85.1 mg/g of total hydroxycinnamic acids (57.4 mg/g of caffeoylquinic acids, mainly 5-CQA) and 20.5 mg/g of total methylxanthines (20.2 mg/g was caffeine). Therefore, the daily consumption of hydroxycinnamic acids and methylxanthines was 510.6 and 123 mg, respectively. Several parameters were analyzed at the beginning and end of the study to assess any differences before and after coffee intake. SDB and DBP, total body fat, WC, TG, HDL-C, fasting glucose, fasting insulin concentration, and IR (HOMA-IR) were analyzed. Then, leptin, resistin, PAI-1, and visfatin were analyzed in plasma samples. This study showed interesting results, particularly regarding blood pressure, blood glucose, and triglyceride levels. The positive effect on TG was more pronounced in the hypercholesterolaemic group. Moreover, with regard to WC, this was mostly decreased in this group. The hypoglycaemic and anti-diabetic effect of coffee is mainly due to the chlorogenic acid, which also improves glucose tolerance and IR. 

Another effect observed was the decrease in adipokines (leptin, PAI-1, and resistin), probably due to the decrease in body fat mass, with the consequent decrease in adipocytes and thus in adipokines. The concentration of visfatin, on the other hand, did not change, in accordance with the non-change in visceral fat (WC) where it is synthesized. Finally, the greatest effect was on blood pressure in both groups (the reduction in SBD and DBP was −5.2 and −5.6 mmHg in the hypercholesterolaemic group and −3.4 and −2.3 mmHg in the normocholesterolaemic subjects, respectively). Although caffeine has an acute pressor effect, chlorogenic acid probably reverses this effect. Therefore, coffee intake may be recommended to healthy and hypercholesterolaemic subjects to prevent MetS, as it produces positive effects on blood pressure, blood glucose, and triglyceride levels. 

A very interesting study about the beneficial effects of yellow tea against MetS was performed by Guohou Wu et al. [122] using leptin receptor knockout (Lepr^−/−^) rats. They divided the rats into two macrogroups: Lepr^−/−^ rats and wild type (WT). In turn, WT rats were divided into WT control group (WT) and WT that were given tea water extract (LWE) (WL). Lepr^−/−^ rats were grouped into the Lepr^−/−^ control group (KO) and the Lepr^−/−^ with LWE-treated group (KL). Different parameters before and after LWE intake were evaluated, such as blood glucose, glucose tolerance, totChol, triglyceride, fatty acids, and weights of liver, inguinal, and epididymal adipose tissues. LWE supplementation decreased body weight in Lepr^−/−^ rats at 14 weeks of intervention and greatly reduced fat mass gain and increased lean mass. After a 24-week experiment, the rats were sacrificed, and liver tissue were collected, showing higher weights of liver, inguinal, and epididymal adipose tissues in the Lepr^−/−^ group than in WT rats. LWE intake significantly reduced the weights of the liver, inguinal, and EAT compared to the Lepr^−/−^ control group. Furthermore, it has been shown that LWE reduced the area of adipocytes in the inguinal pad and epididymal pad of Lepr^−/−^ rats. Regarding lipid profiles in serum and liver of Lepr^−/−^, LWE supplementation decreased the concentrations of TG, TC, and LDL-C in the liver tessues of Lepr^−/−^ rats, thus improving serum and liver lipid profiles to attenuate MetS in Lepr^−/−^ rats. Comparing the KO and KL groups, lower blood glucose and serum insulin values were recorded in the KL group, in which the HOMA-IR index also improved. An oral glucose tolerance test (OGTT) was conducted, and an increase in the AUC of the OGTT was observed in Lepr^−/−^ rats, suggesting severe glucose intolerance; these results indicated that LWE improved IR and glucose tolerance in Lepr^−/−^ rats. Large lipid vacuoles were observed in the liver tissues of Lepr^−/−^ rats, indicating development of the condition of fatty liver. In the LWE-treated Lepr^−/−^ group, more normal hepatic cells than in Lepr^−/−^ rats were observed, demonstrating that consuming LWE remarkably alleviated fatty liver and protected hepatic injury in Lepr^−/−^ rats. Furthermore, liver lipogenesis was examined in this study; LWE intake reduced the expression of several genes involved in lipogenesis, such as sterol regulatory element-binding transcription factor 1 (SREBP1), PPARγ, acetyl-CoA carboxylase α (ACCα), fatty acid synthase (FAS), and diacylglycerol-O-Acyltransferase 1 (DGAT1). In addition, LWE administration increased the expression of Sirtuin 6 (SIRT6), an enzyme involved in glucose and lipid metabolism. 

Furthermore, the expression levels of SREBP1, FAS, and DGAT1, which are key enzymes controlling hepatic lipogenesis, were significantly higher in the Lepr^−/−^ rats than in the WT rats, which indicated that liver lipid synthesis was significantly enhanced in Lepr^−/−^ rats in comparison with WT rats. In contrast, the Lepr^−/−^ rats with LWE intervention showed a significant reduction in the protein expression levels of SREBP1, FAS, and DGAT. The gut microbiota was also analyzed since correlations were seen between MetS and microbial dysbiosis. LWE treatment has been reported to significantly increase the diversity of gut microbiota in comparison with the Lepr^−/−^ control group rats (*Ruminococcaceae*, *Faecalibaculum*, *Intestinimonas*, *Alistipes*, *Odoribacter*, *Akkermansia*, and *Veillonellaceae*). Additionally, LWE intervention significantly increases the level of short-chain fatty acids SCFAs in Lepr^−/−^ rats, and these changes could be associated with changes in the gut microbiota after taking LWE. These results revealed that LWE attenuated MetS of Lepr^−/−^ rats via the reduction of hepatic lipogenesis through the SIRT6/SREBP1 pathway and the modulation of gut microbiota.

### 5.4. Fruits

Several epidemiological studies on the association between fruit and vegetable consumption and the risk of MetS have been performed and largely reported in the literature [123,124,125].

Berries are a variety of small, pulpy, edible fruit consumed both as fresh products and processed foods that represent an important source of a wide range of beneficial substances. Polyphenols such as flavonoids (flavonols, flavanols, and anthocyanins), phenolic acids (hydroxycinnamic and hydroxybenzoic acids), tannins (proanthocyanidins, ellagitannins, and gallotannins), stilbenoids, and lignans are reported in berries [126]. The effect of cranberry juice consumption on metabolic and inflammatory biomarkers in patients with MetS was assessed in a study conducted by Simão et al. [127]. After consuming 700 mL/day cranberry juice containing 0.4 mg folic acid for 2 months, the levels of homocysteine and adiponectin were measured in two groups recruited for the study: the control group (*n* = 36) and cranberry-treated group (*n* = 20). The results showed an increase in adiponectin (*p* = 0.010) and a decrease in homocysteine (*p* < 0.001) levels in comparison to controls. The consumption of cranberry juice was able to improve some cardiovascular risk factors. Aboonabi et al. [128] investigated the atheroprotective effects of anthocyanins on 55 Australian patients with MetS (aged 25–75 years) after supplementation of 320 mg anthocyanin supplements twice daily for 4 weeks. This anthocyanin supplementations corresponds to 100 g of fresh bilberries. Anthocyanin supplementation for 4 weeks significantly decreased cardiometabolic risk factors and registered a reduction in triglyceride (by 24.9%, *p* < 0.05), LDL-C (by 33.1%, *p* < 0.05), and serum fasting blood glucose (by 13.3%, *p* < 0.05). 

In the last decade, interesting investigations have highlighted the beneficial effects of natural antioxidants and their potential capability in managing MetS. Besides berries, the effect of bergamot, a yellow citrus fruit containing various phytochemicals, flavonoids, and other health-promoting compounds, was also reported in the literature [129]. Bergamot possesses a phytochemicals profile that can be considered unique in its various forms. In particular, it differs from other citrus fruits in the composition of its flavonoids and glycosides. Bergamot contains a considerable amount of neohesperidin, neoeriocitrin, rutin, and naringin, as reported by Salerno et al. [130]. Mollace et al. [131] investigated the bioactivities of bergamot-derived polyphenolic fraction on LDL-C, blood totChol, TG, and blood glucose. A group of 237 patients suffering from isolated or mixed hyperlipemia either associated or not with hyperglycemia were orally given bergamot extract for 30 consecutive days. The consumption of bergamot extract reduces total and LDL-C levels and TG levels along with a concomitant significant decrease in blood glucose. The effect of a combined nutraceutical formulation containing bergamot extract, phytosterols, vitamin C, and chlorogenic acid from dry artichoke extract was investigated in another clinical trial on 90 overweight dyslipidemic subjects. Ninety patients were consecutively enrolled and randomized to receive high-dose (*n* = 30), low-dose (*n* = 30), or placebo (*n* = 30) treatment. After treatment, all active-treated groups experienced a significant improvement in TG and in low-density lipoprotein cholesterol versus baseline and placebo treatments [132].

Nuts are another food category containing an optimum chemical composition able to prevent MetS. Specifically, walnuts stand out for their high PUFAs content, which is aligned with cholesterol-lowering effects that contribute to a reduced risk of coronary heart disease [133]. Hwang et al. [134] investigated whether regular walnut consumption positively changes heart-health-related parameters and how walnut intake impacts MetS. This study involved 210 screened volunteers and demonstrated that daily walnut consumption for 16 weeks improved MetS status. In particular, significant improvements in HDL-C, fasting glucose, HbA1c, and adiponectin after walnut intake compared to the control intervention were observed.

### 5.5. Legumes

Legumes represent a sustainable and inexpensive source of nutrients. In several areas of the world, legumes are valued as a meat alternative. They are a good source of proteins, complex carbohydrates, essential amino acids, and phytochemicals including flavanols, flavan-3-ols, anthocyanins, tocopherols, condensed tannins, isoflavones, and lignans. The consumption of legumes has also been reported to be associated with numerous beneficial health attributes [135]. 

Reverri et al. [136] assessed black bean’s contribution in attenuating postprandial metabolic, oxidative stress, and inflammatory responses through a randomized, controlled, crossover trial on 12 patients with MetS. Participants consumed one of three meals: black bean, fiber matched, and antioxidant capacity matched, supplemented with 300 mg grape seed extract. Blood collection was performed before and five hours postprandially. The addition of black beans in a typical meal attenuates postprandial insulin and moderately enhances postprandial antioxidant endpoints in adults with MetS.

The effects of soy consumption on components of MetS, plasma lipids, lipoproteins, IR, and glycemic control in postmenopausal women with MetS were evaluated by Azadbakht et al. [137]. Participants were randomly assigned to consume a control diet, a soy-nut diet, or a soy-protein diet, each for 8 weeks. A comparison of these diet groups showed a significant difference between the control and soy-protein diets regarding LDL-C, totChol, and fasting insulin. Short-term soy-nut consumption improved glycemic control and lipid profiles in postmenopausal women with the MetS. Ruscica et al. [138] conducted a randomized, parallel, single-center study to evaluate how the substitution of animal-protein-based food with 30 g/day soy protein affects MetS. The inclusion of whole-soy foods in a lipid-lowering diet significantly improved a relevant set of biomarkers associated with cardiovascular risk. At the end of the treatment period, TC, LDL-C, and non-HDL-C in the soy food group versus the control group were significantly reduced.

### 5.6. Cereals

Cereals, grains, and tubers represent the most common staple food worldwide. The principal cereals are wheat, rye, rice, barley, millet, and oats. The consumption of the different cereals is not the same worldwide and depends on several factors, such as what plants are native to a region, traditions, and habits. However, the major constituents of the six kinds of cereal are fairly uniform: starch is the main available carbohydrate, proteins are between 7 and 15% (although a limited content in some essential amino acids), mono and polyunsaturated fatty acids are prevalent, and minerals and trace elements decrease from outer to inner cells.

Several studies have been published attempting to demonstrate the role of different type of cereals with the development of MetS. The major evidence is that whole-grain intake is favourably associated with metabolic risk factors T2D and CVD, whereas refined-grain intake is positively associated with the risk of MetS [139,140,141,142,143,144]. However, some results are still controversial. 

In 2014, Song et al. [140] published a cross-sectional study in which the sources and types of carbohydrates were differentially associated with MetS according to sex in the Korean adult population. The relationship between MetS prevalence and dietary carbohydrate intake (total grains, refined grains, and white rice) in terms of total carbohydrate, energy from carbohydrates, dietary glycemic index, and dietary glycemic load was investigated, and results showed the percentage of energy from carbohydrates in men and intake of refined grains, including white rice, in women was associated with MetS.

A randomized, controlled two-center intervention study was carried out among Italian and Finn adults with MetS to investigate the effects of whole-grain intake on glucose and insulin metabolism in response to 12-week diet based on consumption of rye and whole wheat compared with a diet containing the same amount of refined cereal foods [145]. Biometrical parameters (mean body weight, BMI, WC, fat mass and lean mass, SBP and DBP), fasting plasma concentration of glucose, lipids and insulin, and peripheral insulin level were assessed before and at the end of the intervention. However, no changes were observed between the groups and compared with the baseline, thus concluding that whole-grain cereal foods consumption does not affect peripheral insulin sensitivity. The year after, the same research group published another work on the influence of whole-grain-based or a refined-cereal-based diet on postprandial glucose, insulin, and lipid metabolism in individuals with MetS [146]. The authors carried out the same intervention study, but biochemical parameters were also evaluated 3 h after a lunch. Indeed, changes in postprandial insulin and triglyceride levels after fasting were reduced (29 and 43%, respectively) in the whole-grain-diet group compared to the refined-cereals-diet group, but no changes in glucose levels were found, suggesting a possible effect of whole grain at the liver level.

More recently, a cohort study carried out among healthy people from the northern and southern regions of China found a positive association between the intake of wheat and its products and a risk of MetS, while a negative relationship between rice consumption and MetS was found in southern China [147]. However, regional disparities regarding other foodstuffs were not taken into account, thus affecting the results.

In 2020, a 10-year cohort study with a follow-up every 2 years was published, involving a middle-aged and older Korean population without MetS and with the aim of investigating a possible association between different grain subtype and grain combination with the risk of MetS [148]. At the end of the study, 38.8% of the participants developed MetS. Results were in accordance with the hypothesis that whole-grain consumption was associated with a lower risk of MetS; likewise, a combination of a higher amount of whole grain, a lower amount of refined grain, but higher refined grains and the combination of lower whole grains and lower refined grains was associated with a higher risk of MetS.

Recently, a first case-control study investigating the association between plasma 3-(3,5-Dihydroxyphenyl)-1-propanoic acid (DHPPA), a biomarker of whole-grain wheat and rye intake, with MetS risk in a Chinese population was published [149]. DHPPA was measured by LC-MS/MS analysis, and higher concentrations were found in individuals with lower odds of MetS, thus confirming the inverse correlation between whole-grain consumption and MetS risk. 

Few studies have been carried out focusing on rice matrix. An in vitro study aimed at investigating the role of rice proteins on triglyceride metabolism, which led to an improvement of body weight and adiposity and thus rice’s consideration as important matrix for the prevention and treatment of MetS [150]. Two different rice proteins were isolated, i.e., RP-A and RP-E, and their effects were investigated in rats fed cholesterol-enriched diets for two weeks in comparison to casein as control. The cholesterol-enriched diet was carried out for two weeks, and at the end, rats belonging to the group of RP-A and RPE treatment showed a significantly reduced body weight gain, plasma glucose and lipid levels, and hepatic lipids accumulation compared to the control. Moreover, analyses on several enzymes involved in the regulation of fatty acids synthesis showed reduced activities of fatty acid synthase, glucose 6-phosphate dehydrogenase, and malate dehydrogenase. However, few significant differences were found comparing the RP-A and RP-E treatment groups. These findings suggested a possible role of rice proteins in the lipogenesis and lipolysis, resulting in triglyceride-lowering action as well as the potential of anti-adiposity.

### 5.7. Olive Oil

Olive oil provides monounsaturated fat, which lowers totChol and LDL-C levels [151]. In olive oil, the content of polyphenols depends on olive ripeness, agronomic factors, and extraction technology and ranges from 50 up to 1000 mg/kg. Phenolic compounds occur as phenolic acids, oleuropein derivatives, and flavonoids including oleuropein, tyrosol, hydroxytyrosol, and oleocanthal. Particularly, hydroxytyrosol, an oleuropein hydrolysis-derivate, has the strongest antioxidant effect of the polyphenols reported in olive oil [152].

Violi et al. [153] tested the effect of extra virgin olive oil on post-prandial glycemic and lipid profiles through a cross-over design study conducted on 25 healthy subjects. This is the first study demonstrating that a Mediterranean-type meal supplemented with EVOO has a beneficial effect on postprandial glycemic and lipid profile by decreasing blood glucose, LDL-C, and ox-LDL. Two hours from lunch, in subjects given a meal containing corn oil, the blood glucose, insulin, GLP1, GIP, DPP-4 concentration, and DPP-4 activity increased significantly. The effects of olive and fish oil on cardiovascular risk factors and oxidative stress in patients with MetS were investigated by Venturini et al. [154]. Patients enrolled (*n* = 121) in the experimental study were divided into four groups: control, fish oil group, extra-virgin olive oil group, and fish oil and extra-virgin olive oil group. The main findings were that extra-virgin olive oil administered concomitantly with fish oil improved lipid metabolism, decreased prooxidant state, and increased antioxidant defences.

### 5.8. Omega 3 Long-Chain Polyunsaturated Fatty Acids and Fish Products

Long-chain PUFAs represent essential nutrients in any healthy balanced diet. The two major classes of PUFAs are the omega-3 and omega-6 fatty acids. Like all fatty acids, PUFAs consist of long carbon chains with a carboxyl group at one end of the chain and a methyl group at the other. Omega-3 fatty acids (ω-3s) have a carbon–carbon double bond starting at the third carbon from the methyl (ω) at the end of the chain. Several different ω-3s exist, but the most frequent and abundant in foodstuffs are three: ALA, containing 18 carbon atoms, and two long-chain fatty acids, namely EPA and DHA, containing 20 and 22 carbon atoms, respectively. Ω-3s are present in certain foods such as fish and other seafood (especially cold-water fatty fish such as salmon, mackerel, tuna, herring, and sardines), nuts and seeds (such as flaxseed, chia seeds, and walnuts), plant oils (such as flaxseed oil, soybean oil, and canola oil), and fortified foods (such as certain brands of eggs, yogurt, juices, milk, soy beverages, and infant formulas). Ω-3s play structural (as components of the phospholipids of cell membranes), functional (by producing eicosanoids), and energy (through their oxidation) roles in the body. Beyond their nutritional role, much evidence has highlighted the effects of consuming ω-3s in reducing the risk of several chronic diseases such as CVDs [155], inflammatory diseases, Alzheimer’s disease, dementia, and type 2 diabetes [156]. Among these biological implications, different original articles, reviews, and meta-analyses [157,158] investigated the association between dietary and circulating ω-3s with MetS risk although results are still inconsistent. Moreover, the associations between ω-3s levels and MetS risk seemed to be different between Asian and American/European populations. Some discrepancies are related to genetic differences between these ethnic groups, involving the expression of genes for inflammation, lipid metabolism, energy utilization, and insulin signal transduction. Indeed, a recent study reported that Asian individuals have a lower-risk allele frequency than European ones. Other studies correlated these significant differences to the variations of fatty acid desaturase gene in a Mediterranean population with MetS [159,160,161]. The fatty acid desaturase gene plays a pivotal role in the metabolism of fatty acids and thus in determining the serum PUFAs level. This topic is rather controversial since, although some studies found no association between dietary ω-3s and prevalence of MetS [162], others reported higher circulating ω-3s levels in control cases with respect to patients with MetS [163,164,165,166]. However, studies regarding data directly linking ω-3s intake to the risk of MetS are sparse. The most recent epidemiological studies, either cross-sectional, cohort, or follow-up, highlighted a possible role of ω-3s in preventing MetS in different groups of populations (Asian, European, American). In 2014, Zaribaf et al. [167] reported a cross-sectional study in 420 Iranian female adults (>30 years old) based on a dish-based semiquantitative food frequency questionnaire (FFQ) over the previous year. The prevalence of MetS was 8.2%, showing that increased fish intake was independently related to the lower odds of MetS and its feature. Similar results were obtained by Mirmiran et al. [168], who carried out a based cohort study and a 3.6-year follow-up in 3382 Iran adults (2198 female and 1184 male) aged 19–55 years. The population was divided into three groups based on the amount of fish consumption frequency/week (<30, 30–45, and >45 g/week), and the incidence of MetS was 13.1%. Results showed a lower incidence of MetS in the group characterized by a higher fish consumption (>45 g/week) compared to the group of lower consumption (<30 g/week). In 2012, the same research group attempted to find a possible association between the ω-6/ω-3 PUFA ratio intake and the prevalence of MetS [169] in 2451 Iranian adults. However, although intakes of ω-3 PUFAs (EPA and DHA) were associated with a lower risk of high serum triacylglycerol concentrations regardless of the background intake of ω-6 PUFAs, no correlation was found between the ω-6/ω-3 PUFAs ratio and a decrease in the prevalence of the MetS. The role of fish and/or ω-3 PUFAs in preventing MetS in an American population was investigated by Lai et al. [162] in 2013 and by Kim and co-workers [170] in 2016. The first work was a cross-sectional study carried out on 4941 subjects aged 52.1 ± 13.9, but no association between dietary ω-3 PUFAs and prevalence of MetS was found [162], whereas in the second one, a prospective cohort study with a 25-year follow-up carried out on 4356 subjects from 18 to 30 years old, the ω-3 PUFA intake was inversely associated with the incidence of MetS in a dose–response manner. Results showed a 46% lower incidence of MetS for subjects in the higher quintile of ω-3s intake compared with those in the lowest quintile [170]. 

One more study that supports the impact of fish/ω-3 PUFA on MetS risk involved 1520 Korean adults between 30 and 65 years old. The data obtained suggested that fish consumption (range 18–93 g/day) could maintain blood LDL-C and blood totChol concentrations at the optimal level in subjects with prediabetes or with metabolic risk factors [171].

In addition, other epidemiological studies on European populations corroborate this hypothesis. In particular, Tørris et al. [172] performed a cross-sectional study in 2016 [173] and a 13-year follow-up study in 2017 involving a large sample from a Norwegian population (23,907 subjects), finding that fatty and lean fish consumption influences MetS risk differently, possibly also related to age. Indeed, a lower risk of having MetS among participants aged 60–70 years who consumed fish once a week or more was found compared to those consuming fish less than once a week [172]. Moreover, in the second study, lean fish consumption seems to have a greater beneficial effect on the various components of MetS when compared to fatty fish [173]. Limitations of the reported studies might rely on the applied methodology, which is based on the administration of an FFQ. Indeed, the FFQ might have led to (i) an overestimation or underestimation of dietary ω-3s, (ii) the use of a single FFQ, which may not fully capture dietary habits over time, and (iii) the generalizability of findings to non-White populations. Beyond epidemiological observational evidence, studies involving interventions targeting the relationship between ω-3/fish intake and MetS incidence are still lacking. A study carried out on high-carbohydrate-, high-fat-induced MetS male rats pointed out differences between ALA and EPA/DHA effects [174]. Although all the three ω-3PUFAs reduced inflammation in both the heart and the liver and cardiac fibrosis and hepatic steatosis, EPA and DHA increased sympathetic activation, reduced the abdominal adiposity and total body fat, and attenuated IR, dyslipidemia, hypertension, and left ventricular stiffness but not glucose tolerance. Moreover, interesting results were obtained in a randomized controlled trial involving 87 postmenopausal women with MetS [175]. The dietary intervention involved the supplementation of 900 mg of ω-3s/day for six months and resulted in a further decrease in TG and blood pressure as well as an improvement in IR and inflammatory markers, which are important components of MetS. In 2014, Lee et al. [176] carried out an 8-week, randomized, single-blind, parallel intervention study aiming at comparing the impact of three PUFA-based supplements (corn oil, a botanical oil combination, and fish oil) on the levels of serum fatty acids and other serum lipids (TG and total, HDL-C, and LDL-C), markers of inflammation (leptin and CRP), as well as glucose regulation (glucose and HbA1c) in 59 subjects with early-stage type 2 diabetes or MetS. Results showed that supplementation with botanical oil significantly lowered total and LDL-C levels, whereas fish oil reduced serum TG and HbA1c and increased HDL-C, and the selected markers of the corn oil group were almost similar to the control (pre-treatment). One more double-blind, randomized intervention consisting of a 24-week, high-intensity training alone or combined with ω3s and oleate supplementation was carried out on 36 subjects with MetS. Data showed reduced metabolic (i.e., insulin sensitivity) and cardiovascular (HDL-C, CRP) risk factors in subjects who followed a diet supplemented with ω-3s plus oleate supplementation [177].

The effect of white fish intake on cardiovascular risk factors (in terms of serum fatty acid profile and some MetS features (WC, blood pressure, and fasting blood glucose), LDL-C and CRP, and IR) were evaluated in subjects with MetS by Vazquez et al. [178] through a multicenter, randomized crossover, clinical trial. In this study, 273 patients with MetS followed an 8-week, only-one dietary intervention characterized by 100 g/day of white fish (Namibia hake) with advice on a healthy diet. A significant lowering effect was found in the intervention group regarding WC, DBP, and serum LDL concentrations, whereas serum EPA and DHA fatty acids concentrations were raised as compared to control group (patients with MetS with advice on healthy diet avoiding fish or seafood for the 8-week intervention).

### 5.9. Polyphenols

Bioactive compounds are secondary metabolites produced by plants or found in foods that are able to modulate metabolic processes. Research on phytochemicals showed their possibility as therapeutic and preventive agents against conditions such as inflammation, oxidative stress, dyslipidemia, and IR. Polyphenols are one of largest classes of naturally occurring bioactives secondary metabolites generally involved in defence mechanisms against ultraviolet radiation or pathogens. More than 8000 polyphenolic compounds have been identified in various plant species [179].

The term polyphenols includes several classes of compounds with a common chemical structure: they are benzene derivatives with one or more hydroxyl groups associated with the ring [180]. This structure allows these compounds to actively function as scavengers to stabilize free radicals, reducing agents, chelators of pro-oxidant metals, and quenchers for the formation of singlet oxygen [181]. 

Polyphenols are divided into two main classes: flavonoids and non-flavonoids (phenolic acids, stilbenes, and lignans) according to the number of phenolic rings and the structural elements that bind these rings. The structural arrangement, polymerization degree, and conjugation with other phenolics play an important role in the bioavailability and absorption of dietary polyphenols [182].

#### 5.9.1. Flavonoids

Flavonoids are a group of polyphenolic compounds found naturally in fruits, vegetables, and beverages (coffee, tea, and wine). Their distinctive beneficial effects on MetS are primarily due to structural differences. The basic structure of flavoinoids is characterized by a central skeleton composed of 15 carbon atoms and, more precisely, three rings: two benzyl rings designated as A and B and a heterocyclic ring designated as C. From this general structure, flavonoids are sub-divided into six classes according to their chemical structure: flavones, flavonols, isoflavones, flavanones, flavanols (catechins and proanthocyanidins), and anthocyanins [183].

The positive effect on MetS resulting from the intake of flavonoids has attracted the attention of many researchers. Recently, Grosso et al. [184] showed how the intake of flavonoids reduces the risk of MetS. However, why do flavonoids seem to be so efficient in preventing MetS? The anti-inflammatory and anti-oxidative effects are hypothesized to be the main mechanism against MetS [184,185]. Furthermore, some flavonoids are known to increase endothelial nitric oxide synthase (eNOS) expression and thus NO production [186]. This compound released by endothelial cells is extremely important, as it regulates wall tone and relaxation response. A decrease in NO production leads to a reduction in vasodilation, which in turn could lead to CVD. The anti-inflammatory and anti-oxidative effects also protect endothelial cells from the dysfunction that can lead to vascular regulation disease [186,187]. Apigenin and narinenin, two of the major exponents of the class of flavonoids (flavone and flavone, respectively), decrease the incidence of endothelial dysfunction by decreasing protein kinase C ßII (PKCßII) phosphorylation and reactive oxygen species (ROS) production in endothelial cells exposed to high glucose levels [188]. Furthermore, it has been shown that apigenin reduces high-glucose-increased apoptosis, Bax expression, caspase-3 activity, and nuclear factor kappa-light-chain-enhancer of activated B-cells (NF-κB) phosphorylation in endothelial cells. Due to its ability to inhibit ROS production and caspase-3 activity, apigenin prevents lipopolysaccharide (LPS)-enhanced apoptosis [189]. These properties make apigenin an excellent compound able to stabilize mitochondrial function and prevent endothelial dysfunction in inflammatory states.

Several studies have shown how the intake of these six classes of flavonoids reduces the risk of CVD and stroke [190]. Interestingly, 60,289 women and 38,180 men (mean age 69 and 70 years, respectively) responding to a survey on dietary and lifestyle habits showed how the intake of flavonoids was associated with a decrease in the incidence of CVD [191]. Furthermore, a study conducted on 43,880 healthy men showed that a diet rich in anthocyanins and flavanones decreases the risk of CVD [192]. An interesting study was conducted on 774 elderly Dutch men (aged between 65 and 84 years); they were subjected to consumption of catechin (amount of 15.2 mg/day) obtained from different plant sources. After 25 years, 329 men were seen to have died from CVD, 148 from coronary heart disease (CHD), and 72 from stroke. However, the risk for death from CVD and CHD decreased by catechin intake [193]. Long-term consumption of flavonoids was associated with reduced incidence of high blood pressure. For example, epidemiological studies suggest that cocoa-rich products prevent the risk for CVD. In a prospective cohort of 40,574 disease-free French women, it was observed that participants with higher flavonol, anthocyanin and polymeric flavonoid intakes and higher total flavonoid intakes were less likely to develop hypertension [194]. A double-blind, controlled, parallel-arm study showed how flavanol from cocoa intake improved blood pressure and metabolic profile in elderly subjects [195]. Participants consumed high (993 mg), intermediate (520 mg), or low (48 mg) quantities of flavanol from beverages including cocoa flavanols. Improved insulin sensitivity and blood pressure and lipid peroxidation were observed in the high-flavanol-intake group compared with the low-flavanol-intake group. Moreover, a study conducted on 102 hypertensive men for three months demonstrated that consuming 6 or 25 g/day of flavonoid-rich dark chocolate had beneficial effects on blood pressure [98]. The positive effects of dietary flavonoids on cardiovascular health were investigated in 272 Japanese men in a cross-sectional study [196]. Serum levels of isoflavone/equol were analyzed. Equol, a metabolite of the dietary isoflavone daidzein, is produced by the action of gut bacteria in some individuals who are termed as equol producers. It was found that equol producers showed a decreased incidence of coronary artery calcification (CAC). No significant correlations were found between isoflavone and CAC. Flavonoids have shown good activity in counteracting obesity complications; increased intake of flavonols, flavanols, anthocyanins, and flavonoid polymers may be associated with weight maintenance in adults [197].

#### 5.9.2. Chlorogenic Acid

Chlorogenic acid is one of the main phenolic acids and is particularly present in coffee and in different types of fruits. Chlorogenic acid belongs to the hydroxycinnamic acids family; its structure derives from the combination of caffeic acid with quinic acid, held together by an ester bond in C5. For this reason, it is also called 5-O-caffeoylquinic acid (5-CQA). Chlorogenic acid has been shown to be an extremely important compound in preventing and treating MetS given its countless properties such as antioxidant, anti-inflammatory, hypolipidemic, antidiabetic, and antihypertensive.

Huang et al. [198] demonstrated 5-CQA activity against MetS. This in vivo study was conducted on forty male Sprague–Dawley rats divided into four groups: normal diet, high-fat diet, low-chlorogenic acid (20 mg/kg body weight) and high-fat diet, and high-chlorogenic acid (90 mg/kg body weight) and high-fat diet. Chlorogenic acid was dissolved in sterile saline and administered orally by gavage once daily for 12 weeks. Chlorogenic acid suppressed in a dose-dependent manner body and visceral fat weight gain and hepatic free fatty acids induced by high-fat diet. Furthermore, chlorogenic acid has shown beneficial effects against diabetes in both animals and humans. A study performed in an experimental animal model (20 Leprdb/db mice and four C57BL/6 mice as control group) showed interesting activities of chlorogenic acid against diabetes [199]. Mice were treated daily for 2 weeks with vehicle, 250 mg/kg BW metformin by oral gavage, or 250 mg/kg BW chlorogenic acid. Chlorogenic acid inhibited hepatic glucose-6-phosphatase expression and activity, decreased hepatic steatosis, and improved lipid profiles and skeletal muscle glucose uptake, which improved fasting glucose levels, glucose tolerance, insulin sensitivity, and dyslipidemia in the Leprdb/db mice. The anti-obesity and antidiabetic properties of chlorogenic acid have been attributed to its impact on glucose metabolism as well. 5-CQA is able to inhibit glucose absorption in the small intestine by inhibiting glucose-6-phosphate translocase 1 [200], resulting in the reduction of glucose transport in the intestine and glucose release by inhibiting hepatic glucose-6-phosphatase activity [201]. This leads to a decrease in circulating glucose that results in higher consumption of fat reserves as an energy source. The lower insulin activity (due to the decreased blood glucose concentration) leads to a reduction in fatty deposits.

Jin et al. [201] showed that 5-CQA increases adiponectin receptors and phosphorylation of AMP-activated protein kinase (AMPK) in late-diabetic mice. They studied thirty-two female C57BL/BKS mice divided into four groups (two control groups and two chlorogenic acid groups). Animals in the chlorogenic acid groups were treated once daily with chlorogenic acid (80 mg/kg BW) for 12 weeks by lavage, while controls were given phosphate-buffered saline (PBS). Compared to the control group, the percentage of body fat, fasting plasma glucose, and HbA1c significantly decreased (*p* < 0.05) in the chlorogenic acid groups. Reduction of glucose-6-phosphatase activity and increase in adiponectin receptors, adiponectin, and AMPK phosphorylation have been associated with a decrease in fasting glucose, HbA1c, TG, cholesterol, and hepatic steatosis and increased insulin sensitivity and glucose tolerance. Chlorogenic acid has shown interesting activity against dyslipidaemia. Moreover, an in vivo study in 2013 analyzed how rats fed a cholesterol-rich diet responded to increasing doses of chlorogenic acid [202]. Forty mice were divided into four groups, two of which received the following doses of chlorogenic acid: 1 mg/kg BW/day and 10 mg/kg BW/day (together with a diet rich in cholesterol). The frequency of administration was one dose per day for 28 days. The third group (normal diet) and the fourth group (cholesterol-rich diet) were treated just with distilled water. High doses of 5-CQA were found to significantly reduce total and LDL-cholesterol and increase HDL-C.

We have already mentioned that endothelial cells play an important role when it comes to MetS. Healthy men and women (*n* = 23) were recruited to a randomized, double-blind, placebo-controlled, crossover trial [203]. Subjects were treated with water (control) and 400 mg of 5-CQA dissolved in 200 mL of low-nitrate water. Data analysis showed a decrease in both SBP and DBP blood pressure (−2.41 and −1.53 mmHg) in subjects treated with chlorogenic acid, while NO status and endothelial function were not significantly influenced. Thanks to these properties of chlorogenic acid, there is a growing awareness of how fruits rich in this metabolite can “protect” humans from diseases such as CVDs and hypertension. 

### 5.10. Curcumin

Curcumin is a constituent of traditional medicine as well as a bioactive compound contained in turmeric [204]. Turmeric (*Curcuma longa*) is a plant native to India and cultivated in several other parts of the world, including Southeast Asia, China, and Latin America [205]. Chemically speaking, curcumin (1,7-bis-(4-hydroxy-3-methoxyphenyl)-hepta-1,6-diene-3,5-dione) is a lipophilic polyphenol and is known for its therapeutic potential primarily as an anti-inflammatory and anti-diabetic agent [206,207]. Mechanisms of action of curcumin also include induction of apoptosis, inhibition of proliferation, and suppression of a variety of cell signaling pathways [208]. Considering that turmeric’s nutritive constituents make it a potential candidate for obesity treatment, the effects of curcumin on adipogenesis and, in particular, on pre-adipocytes were analyzed in a cell-type study [209]. To explore the dose and time effects of curcumin, 3T3-L1 pre-adipocytes were seeded in plates and treated at different curcumin concentrations (0, 10, 15, 30, and 50 μM) and at different exposure times (0, 24, 48, and 72 h); after ten days, more than 95% of the pre-adipocytes had differentiated into adipocytes. Cell viability was determined using the MTT test, showing that cell survival was not affected by treatment with 10 or 20 μM curcumin, but cell death was induced by 30 or 50 μM curcumin, and the cytotoxic effect was dose/time-dependent. What is evident from the study is that high-dose curcumin induces apoptosis of pre-adipocytes in a time- and dose-dependent manner via caspase pathways; low-dose curcumin inhibits adipocyte differentiation by altering the expression of cell cycle regulators, reducing mitotic clonal expansion (MCE), downregulating PPARγ and C/EBPα expression, preventing β-catenin under regulation, and decreasing lipid accumulation. These results suggest that curcumin supplementation could be an effective strategy to treat/prevent the development of obesity by curcumin-induced reduction of preadipocyte number and adipocyte fat mass.

Curcumin reduces interleukin-6 (IL-6) and TNF-α levels and induces the expression of adiponectin, the most important anti-inflammatory agent secreted by adipocytes. The clinical activity of curcumin on these effects was evaluated in patients with obesity suffering from MetS [210]. The randomized study was conducted on 44 Caucasian patients aged between 18 and 70, with a BMIbetween 25.0 and 29.9, and diagnosed with MetS [6]. For the first thirty days, the study participants followed a lifestyle change (food consumption, physical activity, and emotional reactions). This was followed by the treatment phase, and 22 of the participants were supplemented twice daily for one month with a food supplement formulated to be enteric-coated and containing 800 mg/dose/day of *Curcuma longa* extract (95% curcumin) complexed with sunflower phospholipids (20% phosphatidylserine) and blended with 8 mg/dose/day of piperine from *Piper nigrum* extract. The other 22 participants represented the control group and were assigned to treatment with a nutritional supplement containing 400 mg/dose/day of pure phosphatidylserine. Weight, fat percentage, WC, hips, and BMI were assessed for each individual. In the curcumin group, the possibility of losing about 1 kg every 10 days was observed, and the benefit of curcumin remained evident when the phosphatidylserine group was taken as a comparison. Thus, the results showed that curcumin supplementation can significantly improve the management of overweight patients in terms of anthropometric measurements and body composition. By complexing curcumin with phospholipids and mixing it with piperine, it was also possible to improve bioavailability and reduce urinary excretion, respectively.

The increased oral bioavailability of curcumin by piperine was also demonstrated in another randomized, double-blind, placebo-controlled study describing the effects of curcuminoids on 117 subjects with MetS [211]. Curcuminoids were administered at a daily dose of 1 g and were co-integrated with piperine (10 mg/day) for eight weeks. Blood samples of the participants were collected at baseline and at the end of the study. The results showed that eight-week supplementation with the bioavailability-optimized curcuminoid–piperine combination caused a significant reduction in the levels of biomarkers of systemic antioxidant capacity (SOD), lipid peroxidation (MDA), and inflammation (CRP). Hence, a significant improvement in lipid profile indices, total and low-density lipoprotein cholesterol, high-density lipoprotein cholesterol, and TG was observed.

The anti-hyperglycaemic and insulin-sensitising effect of curcumin was evaluated in male rats with T2D [212]. Rats were divided into 10 groups representing two regimens of the study: the protection regimen, in which treatments were administered for the entire period of the experiment (60 days) together with a high-fat diet (HFD), and the treatment regimen, in which treatments were administered for 15 days after the 60-day feeding on a high-fat diet (total 75 days). In the two treatment regimens, the groups were divided into the control group fed a normal diet, group fed HFD, group supplemented with 80 mg/kg/day curcumin, group treated with 1 mg/kg/day rosiglitazone, and group treated with 80 mg/kg/day curcumin + 1 mg/kg/day rosiglitazone. Curcumin, by attenuating TNF-α levels, was first shown to improve glucose tolerance and lipid profile in HFD-fed rats compared to untreated HFD-fed rats. Furthermore, the effects of curcumin may be comparable to those of the anti-hyperglycaemic drug rosiglitazone, showing its potential role in T2D. The effects of curcumin and ω-3 polyunsaturated fatty acids on circulating levels of GSK-3β in adults at high risk of developing T2D (with impaired fasting glucose or impaired glucose tolerance) were evaluated in a double-blind, randomized, controlled trial [213]. Participants were divided into four interventions: (PL) placebo, (CC) curcumin tablets (180 mg/day), (FO) 1.2 g DHA + EPA, and (CC + FO) dual-action tablets of 180 mg curcumin plus two capsules providing 1.2 g DHA + EPA. Anthropometric measurements were taken, and each participant was asked about diet, physical activity, and medical history. After 12 weeks of curcumin supplementation, circulating levels of GSK-3β were significantly lower in the curcumin group [214]. Insulin sensitivity was significantly improved in the CC supplemented group compared to PL. FO and CC-FO tended to improve insulin sensitivity, but the difference did not reach significance. Thus, this study failed to demonstrate complementary benefits of curcumin and ω-3 polyunsaturated fatty acids on glycemic control. In contrast, Bateni et al. [215] showed that consumption of nano-micellar curcumin has no considerable effect on glycemic indexes and IR. In a randomized, double-blind clinical trial, 50 patients were enrolled to receive 80 mg/day of nanocurcumin or placebo for 12 weeks. Analysis between the two groups showed reduced mean change in triglyceride levels and pancreatic β-cell function, but there were no significant differences in anthropometric measurements, blood pressure, and biochemical factors. Considering that antipsychotics induce altered glucose and lipid metabolism, the potential ability of curcumin to attenuate risperidone-induced metabolic dysfunction was evaluated [216]. Forty-eight female mice (C57BL6) were divided into four groups and randomized according to weight. One group was treated once a week with risperidone (intraperitoneal injection of 12.5 mpk) for 22 weeks; one group was treated with risperidone and curcumin (0.05% Biocurcumax™); the other two groups were controls. Curcumin reduced risperidone-induced hepatomegaly by suppressing the ability to induce hepatic overexpression of enzymes involved in lipid metabolism (LXRα, FAS, ACC1, LPL, PPARγ, ACO, and SREBP-2); it also reduced glucose intolerance and triglyceridemic in risperidone-treated animals and decreased values of serum markers of hepatotoxicity and the hepatic pro-inflammatory transcription factor NFκB.

### 5.11. Prebiotics and Probiotics 

Probiotics reside in the human gut and modulate gastrointestinal microbiota and immunological responses. Prebiotics are non-digestible oligosaccharides that have a beneficial effect by stimulating the growth or activity of beneficial bacteria in the colon. When combined, probiotics and prebiotics constitute symbiotics.

Recent investigations suggest that manipulation of gut microbiota by probiotics, prebiotics, and symbiotics could be a promising approach for the management of MetS [217]. One hundred and twenty adults aged between 35 and 70 years were enrolled in a randomized, double-blind, parallel-group clinical trial. Forty participants were supplemented with 6 g/day of probiotics containing freeze-dried *Lactobacillus acidophilus, Bifidobacterium bifidum, Bifidobacterium lactis, and Bifidobacter longum* (1.5 × 10^9^ each); forty participants received symbiotics comprising the above-mentioned probiotics plus inulin as a prebiotic; the last forty individuals represented the placebo group. In all groups, percentage of obesity, hyperglycemia, hypertension, hypertriglyceridemia, HDL-C, and tendency to develop MetS was assessed. A reduction in the prevalence of hyperglycemia was observed in the probiotic and symbiotic groups and a decrease in the tendency to develop hypertension in the probiotic group. Considering that MetS can lead to T2D, the use of probiotics and symbiotics could provide an important tool to combat the diseases associated with MetS. In particular, a study aimed to evaluate whether the consumption of the probiotic strain *L. reuteri* V3401, together with recommendations on a healthy lifestyle characterized by low-calorie diet and physical activity, was able to improve the components of the MetS [218]. The randomized, double-blind, crossover study was performed according to the criteria of the IDF. A total of 53 patients was divided into two groups: 28 participants received a capsule containing the probiotic *L. reuteri* V3401 (5 × 10^9^ colony-forming units) for 12 weeks, and 25 participants were given placebo. The subjects were monitored by assessing anthropometric and biochemical characteristics and analyzing inflammatory serum biomarkers. IL-6 and soluble cell adhesion molecule-1 (sVCAM) were modified by probiotic consumption, together with an increase in the phylum of Verrucomicrobia. However, no differences in the clinical features of the syndrome were found between the two groups. Indeed, all subjects included in the study lost weight and improved their metabolic status through a healthy lifestyle that included diet and physical activity.

## 6. Microbiota and Nutrigenetics

Most of the strategies targeted to manipulate human microbiota composition do not seem to produce stable changes in human microbiota, with the exception of fecal transplant (although this practice is still somewhat challenging [219,220]). Diet could be assumed to be one of the most prominent elements altering microbiota composition, explaining up to 50% of its variability, according to some authors [221,222]. There is some evidence that dietary interventions can affect microbiota composition in as few as 1–4 days [223,224]; however, as pointed out earlier, these changes are reversed as soon as individuals return to their previous dietary habits. Standardized prospective studies on humans are needed to confirm this preliminary body of evidence and to promote the use of microbiota modulation for MetS prevention and treatment [225,226]. With reference to nutrigenetics, studies have been looking into how the human circadian system mediates metabolic responses to diet by means of its master regulator, the CIRCADIAN Locomotor Output Cycles Kaput (CLOCK) genes complex. Carrying the TT haplotype in the CLOCK single-nucleotide polymorphism, rs1801260 was associated with reduced insulin levels and HOMA-IR in patients with MetS following a 1-year, low-fat diet but not in those following a Mediterranean diet [226]. Other studies have analyzed the interaction between specific SNPs and dietary patterns. Specifically, APOC3 (Apolipoprotein C3), APOA1(apolipoprotein A1), and MC4R (Melanocortin-4 receptor) polymorphisms increase the risk of developing MetS among patients following a Western dietary pattern; other polymorphisms in APOB (Apolipoprotein B) and TCF7L2 (transcription factor 7 like) are risk factors for MetS in the setting of high saturated fatty acid (SFA) intakes [227].

## 7. Conclusions

MetS is a constellation of CVD risk factors, so treatment of this clinical condition and its components could reduce and hopefully prevent chronic metabolic disorders and CVD death. Commonly, the diagnosis of MetS is based on the measurement of a few simple parameters such as WC, blood pressure, HDL-C, triglyceridemia, and blood glucose. A metabolomic approach using NMR spectroscopy and/or chromatographic technique, can be applied to identify a number of metabolites that evolve with MetS or its individual components. Therefore, these molecular biomarkers could be used to define and predict the condition of patients with MetS.

To date, several nutrients, food components, or a combination of both as well as different dietary patterns have been studied to test their effect and therapeutic potential in MetS treatment. Some of the many existing nutraceutical compounds could be used as supplements in the daily diet due to their easy availability and beneficial properties. Although several nutrients have been shown to counteract the individual MetS components, no specific dietary approach for overall MetS therapy has been studied. Recently, a great deal of interest has been directed toward the role of the gut microbiota and its manipulation by prebiotics/probiotics, which could provide new insights into the pathophysiology of MetS and contribute to the development of new approaches for the treatment of metabolic alterations that lead to MetS. Furthermore, based on the evidence, we can assert that polyphenols intake could contribute to beneficial and protective effects on MetS. To further understand the effects of polyphenols on health, further studies should be undertaken by researchers. Such findings could be effective for the development of polyphenol supplementation strategies to maximize health effects.

## Figures and Tables

**Table 1:** Summary description of studies discussed regarding MetS-related pathologies and predictive methods for diagnosis. The experimental model, method, markers for diagnosis, and metabolites regulation are described.

Experimental Model	Detection Method	Markers for Diagnosis	Metabolites Regulation in MetS or Its Correlated Pathologies	Ref.
Human serum (overweight adults)	NMR	CH_3_ lipids	↑	[47]
		CH_2_ lipids	↑	
		CH_2_-CH= lipids	↑	
		Lactate	↑	
		Alanine	↓	
		Glucose	↑	
		Choline	↓	
Human, Zucker rat, and mice urine (subjects affected by T2D)	NMR	Creatinine	↓	[48]
		*N*-acetyl group (glycoproteins)	↓	
		Allantoin	↓	
		Glutamate	↓	
		Glutamine	↓	
		Histidine	↑	
		BCAA (valine, leucine,	↑	
		and isoleucine)		
		*N*-butyrate	↑	
		Citrate	↑	
		Lactate	↑	
Sprague–Dawley mice urine	NMR	Lactate	↑	[49]
		Acetone/acetoacetate	↑	
		Pyruvate	↑	
Human serum, patients affected by MetS and HUA (hyperuricemia)	NMR	Glutamine,	↓	[50]
		Trimethylamine (TMA)	↓	
		Isoleucine	↓	
		Alanine	↓	
		Lysine	↓	
		Lipids	↑	
		3-Hydroxybutyrate	↓	
		Glutamate	↓	
		Glucose	↑	
		Citrate	↓	
		Proline	↓	
		Glycine	↓	
		Tyrosine	↓	
		Triglycerides	↑	
		1-Methylhistidine	↓	
Human plasma (T2D and obesity)	NMR	BCAAs (branched-chain amino acids)	↑	[51]
		AAAs (aromatic amino acids)	↑	
		Alanine	↑	
		Isoleucine	↑	
		Phenylalanine	↑	
		Tyrosine	↑	
		Glutamate/glutamine	↑	
		Aspartate/asparagine	↑	
		Arginine	↑	
		Tryptophan	↑	
		α-Methyl butyryl carnitine	↑	
		Iso valeryl carnitine	↑	
		α-Hydroxybutyrate	↑	
		Glycine	↓	
		Betaine	↓	
		Acylcarnitine	↑	
		Long-chain FAs	↑	
Five-week-old male Sprague–Dawley rats’ urine	NMR	Acetate	↑	[52]
		Leucine	↑	
		Lysine	↑	
		Glucose	↑	
		Citrate	↓	
		2-Oxoglutarate	↓	
		Hippurate	↓	
		Allantoin	↑	
		Creatinine	↓	
		Trigonelline	↓	
		Tryptophan (TRP)	↓	
		3-Hydroxybutyrate (3-HB)	↑	
		Dimethylamine (DMA)	↓	
		Succinate	↓	
		Acetoacetate	↓	
Human plasma (patients affected by T2D and CVD)	NMR	Valine	↑	[53]
		Leucine	↑	
		Isoleucine	↑	
Human serum (patients affected by obesity, IR, and T2D)	NMR	Isoleucine	↑	[54]
		Leucine	↑	
		Valine	↑	
		Phenylalanine	↑	
		Tyrosine	↑	
		Alanine	↑	
		Histidine	↓	
		Glutamine	↓	
Human serum (IR, glycemia, and T2D)	NMR	Alanine	↑	[55]
		Lactate	↑	
		Pyruvate	↑	
		Tyrosine	↑	
		BCAAs	↑	
		Leucine	↑	
		Isoleucine	↑	
		Valine	↑	
		Phenylalanine	↑	
		AAAs	↑	
Male Sprague Dawley rats urine	NMR	Creatinine	↑	[56]
		Creatine	↑	
		Arginine	↑	
		Aspartate	↑	
C57BL/6J (B6) and leptin-deficient *ob/ob* mice (obese) serum and urine	NMR	Acetoacetate, acetone, citrate, fumarate,		[57]
		2-oxoglutarate, succinate, trimethylamine (TMA), and		
		3-hydroxybutyrate are up-regulated		
		for urine sample and acetoacetate,		
		acetone, succinate, carnitine, VLDL/LDL cholesterol, and		
		TMAO for serum		
Human serum (overweight and patients with obesity and with or without MetS)	NMR	BCAAs	↑	[58]
		AAAs	↑	
		Orosomucoid and fatty acids	↑	
Human plasma (patients affected by MetS)	LC/GC-MS	Hydroxypalmitic acid	↓	[59]
		Cholesterol	↓	
		Sphingosine-1-phosphate	↓	
		Lactic acid	↑	
		Alanine	↑	
		Cysteine	↑	
		Lysine	↑	
		Cystine	↑	
		Glutamic acid	↑	
		Valine	↑	
		Proline	↑	
		Aspartic acid	↑	
		Tryptophan	↑	
		Tyrosine	↑	
		Phenylalanine	↑	
		Urea	↑	
		Uric acid	↑	
		Sorbitol	↑	
Human serum (patients affected by MetS)	NMR	C14:0	↑	[60]
		C16:0	↑	
		C18:0	↑	
		C18:1n-9c	↑	
		C18:2n-6c	↑	
Human plasma (T2D)	LC-MS	14 sphingolipids including ceramides (d18:1/18:1, d18:1/20:0, d18:1/20:1, and d18:1/22:1), saturated sphingomyelins (SMs) (C34:0, C38:0, and C40:0), unsaturated SMs (C34:1, C36:1, and C42:3), and hydroxyl-SMs (C34:1, C38:3) are positively associated with incident T2D		[61]
Human plasma (T2D, obesity and MetS)	LC	Ceramides (d18:1/16:0, d18:1/18:0, d18:1/20:0, d18:1/22:0, and d18:1/24:0) and SMs (d18:1/18:0, d18:1/18:1, and, d18:1/20:0) are associated with obesity and MetS		[62]
		Ceramides (d18:1/24:1) are associated with triglyceride change		
		SMs (C36:0 and d18:0/24:0) are associated with glucose change		
		Ceramides (C18:0, C20:0, and C24:1) are associated with cardiovascular disease and T2D		
Human serum (MetS, T2D, and CVD)	GC-MS	2-Hydroxybutyric acid	↑	[63]
		Inositol	↑	
		D-glucose	↑	
Human urine (MetS)	LC-MS	Metabolites in patients with MetS with respect to control		[64]
		Indole-3-acetic acid		
		Indole-3-acetic acid-O-glucuronide		
		*N*-(indol-3-ylacetyl) glutamine Indole-3-carbaldehyde		
		Hydroxyhexanoycarnitine		
		Indole-3-carboxylic acid		

## Data Availability

Not applicable.

## Supplementary Materials

The following supporting information can be downloaded at: https://www.mdpi.com/article/10.3390/nu15030640/s1, Table S1: Summary list of all foods and food components that play a key role in the prevention and/or treatment of MetS.
