# Supplementary material for: New Advances in Metabolic Syndrome, from Prevention to Treatment: The Role of Diet and Food
Source: Nutrients. 2023 Jan 26;15(3):640. doi: 10.3390/nu15030640 (PMC9921449; doi:10.3390/nu15030640)
Supplement: Supplementary file 1 [file nutrients-15-00640-s001.zip › nutrients-2122182-supplementary.pdf]

Supplementary Material

Inclusion and exclusion criteria

The search was carried out using four electronic databases, MEDLINE (via PubMed), ISI Web of Science, Google Scholar, and Scopus. The following inclusion criteria were established: (a) full texts written only in English; (b) published over the last 10 years; (c) primary research articles; (d) combination of the following keywords: <foods> and <metabolomic syndrome>; <food components> and <metabolomic syndrome> in the title, abstract and keywords; (e) sampling consisting of products intended for human consumption.

Exclusion criteria were: (a) any publication lacking from a peer-review process (reports from public institutions and proceedings of conferences) or different reports from the same study (b) review, systematic review, meta-analysis, or book chapters.

Table S1. Summary list of all foods and food components that play a key role in the prevention and/or treatment of MetS.

Foods/Foods components	Country	Study Design	N° participants	Exposure nutrients/ compounds	Exposure assessment	Treatment duration	Main outcome	Ref.
Seeds<br>Pumpkin seeds	Algeria	*In vivo* study	24 male Wistar rats	• C group (20% casein)<br>• P group (20% casein and purified pumpkin seed proteins by oral at the dose of 1 g/kg/day)<br>• C-HF group (20% casein enriched with 64% fructose)<br>• P-HF group (20% casein, purified pumpkin seed proteins, administered by oral gavage, at the dose of 1 g/kg/day and enriched with 64% fructose).	Body weight, food intake, weight gain, food efficiency, BMI adiposity index, TNF-α and IL-6 levels (anti-inflammatory activity), HOMA-IR, HOMA-β and insulinogenic index	8 weeks	Treatment with pumpkin seeds protein counteracted the fructose alteration by improving glucose, insulin, insulinogenic index, HOMA-IR, HOMA-β, glucose tolerance and AUC, suggesting a protective role on complications associated with MetS.	[115]

Seeds<br>Flaxseed	Iran	Randomized controlled clinical trial	60<br>with MetS	• Group one was given 25 mL/day of flaxseed oil<br>• Group two 25 mL/day of sunflower oil	Coagulation score, total antioxidant capacity (TAC) and inflammatory parameters<br>(IL-6)	8 weeks	Dietary intake of flaxseed oil as the main source of dietary fat could improve the inflammatory status among patients with MetS.<br>No significant changes were observed in serum levels of TAC and coagulation score.	[116]
Plants<br>Garlic *(Allium sativum L.)*	Iran	Double-blind, randomized controlled clinical trial	90	• The first group was given 4 garlic powder tablets per day (400 mg of garlic powder per tablet)<br>• The second group received a placebo (also 4 times a day)<br><br>Patients were asked to take two tablets one hour before lunch and another two one hour before dinner	DBP and SBP, insulin resistance, body mass index, fatty liver, serum insulin index and appetite	3 months	Garlic powder supplementation improved MetS, insulin resistance, FLI, and appetite.	[119]
Plants<br>Artichoke<br>(*Cynara scolymus*)<br>leaf extracts<br>(ALE)	Iran	Double-blind placebo-controlled randomized clinical trial	80<br>patients with MetS of which only 68, however, completed it	The subjects were divided into two groups:<br>• the ALE group that received 1800 mg/day of artichoke leaf extracts distributed in 4 tablets per day<br>• the placebo group that received tablets containing cornstarch, lactose and avicel	Concentration of HDL-C, TG and FBS in serum. Monitoring the levels of glutathioneperoxidase (GPx) and superoxide dismutase (SOD) of red blood cells (RBC) and TAC(Antioxidant activity)	12 weeks	ALE decreased serum<br>ox-LDL level in patients with MetS, with no beneficial effects on other antioxidant indices.	[120]
Plants<br>Ginger (*Zingiber officinale* Roscoe)	New South Wales (Australia)	*In vivo* and *in vitro* study	35<br>rats divide in 5 groups (7 rats for each group) fed a diet rich in fat and carbohydrates (HFHC)	• Group 1 was given a standard diet (control group)<br><br>• Group 2 was HFHC control<br><br>• Groups 3 and 4 were given ginger extracts (100 mg/kg and 200mg/kg per day)<br><br>• Group 5 was treated with metformin (200 mg/kg)	Blood insulin concentration (in vivo study);<br>AMPKa phosphorylation in L6 skeletal muscles cells myotube and mRNA expression of PGC-1a (in vitro test)	10 weeks	Ginger has beneficial effects on modulating glucose metabolism in rats fed a high-calorie diet and suggest that ginger may be effective in preventing the development of metabolic syndrome and type 2 diabetes.	[121]

Nervine plants Green and roasted coffee	Spain	Randomized, controlled, crossover clinical study	52	• 25 normocholestero-laemic (total cholesterol < 200 mg/dL)<br>• 27 hypercholestero laemic (total chol-esterol > 200–240 mg/dL)	Total soluble polyphenols and Methylxanthine were analysed by (HPLC– DAD). SBP and TBP, total body fat and WC, triglycerides and HDL-C, fasting glucose, fasting insulin concentration and insulin resistance (HOMA-IR)	8 weeks + 3 weeks of washout stage + 8 weeks = 19 weeks	Regular consumption of a green/roasted coffee blend may be recommended to healthy and hypercholesterolaemic subjects to prevent MetS, as it produces positive effects on blood pressure, blood glucose and triglyceride levels, being particularly interesting for hypercholesterolaemic subjects as coffee improved the lipid profile.	[122]
Nervine plants Yellow tea	China	*In vivo* study	22 rats divided into two macrogroups:<br>• $Lepr^{-/-}$ rats<br>• wild type (WT).<br>WT rats were divided into:<br>• WT control group (WT)<br>• WT that were given tea water extract (LWE), (WL).<br>$Lepr^{-/-}$ rats were grouped into the $Lepr^{-/-}$ control group (KO) and the $Lepr^{-/-}$ with LWE treated group (KL).	The rats were administered water or LWE (700 mg/kg BW) daily by oral gavage.	Parameters evaluated: blood glucose, glucose tolerance, total cholesterol, triglyceride, fatty acids, weights of the liver, inguinal, epididymal adipose tissues, liver lipogenesis and gut microbiota	24 weeks	LWE supplementation improved MetS in Lepr−/− rats by modulating gut microbiota composition and suppressing hepatic lipogenesis.	[123]

Fruits Cranberry juice	Brazil	Clinical trial	56	• 0.7 litres/days cran-berry juice containing 0.4 mg folic acid	Biochemical biomarkers	2 months	The cranberry-treated group showed: ↑ in adiponectin ↑ in folic acid and a ↓ in homocysteine.	[128]
Fruits Bilberries and blackcurrants	Australia	Clinical trial	28 with MetS	• 320 mg anthocyanin supplements twice dai-ly	Biochemical biomarkers	4 weeks	Consumption of anthocyanin supplements improves in cardiometabolic biomarkers and ↓ reduces the risk of thrombosis.	[129]
Fruits Bergamots	Italy	Randomized, double-blind, placebo-controlled	• 104 patients with hypercholesterolemia. • 42 patients with hyperlipidemia • 59 patients with hyperlipidemia and glycemia	1) oral dose of bergamot extract (500 mg/day; A1, B1 and C1) 2) 1000 mg/day of BPF (A2, B2 and C2) 3) placebo (APL, BPL, and CPL).	Hyperlipide-mic and hyperglycemic blood parameters	1 month	The consumption of bergamot extract: ↓ reduces total and LDL-C levels, ↓ triglyceride levels and by a significant decrease in blood glucose.	[132]
	Italy	Randomized, double-blind,placebo -controlled, three-arm, parallel-group clinical trial	90	2 pills of either active treatment contained: • bergamot extract (120-mg flavonoids/ pill) • 120-mg phytosterols/pill •dry artichoke extract (2-mg chlorogenic acid/pill) • 20-mg vitamin C or placebo	Biochemical biomarkers	8 weeks	After treatment, all active-treated groups experienced a significant improvement in triglycerides and in low-density lipoprotein cholesterol versus baseline and placebo treatments.	[133]
Fruits Walnuts	Korea	Randomized, controlled, crossover study	119	45 g of walnuts/day	Blood samples analysis and plasma lipid extraction	16 weeks	A dietary supplement favorably changed MetS status by increasing the concentration of HDL-C and ↓ decreasing fasting glucose level.	[135]

Legumes Black bean	USA	Randomized, controlled, crossover trial	36<br>• black bean meal (n=12)<br>• fiber-matched meal (n=12)<br>• antioxidant capacity matched meal (n=12)	• black bean meal consisted of the moderate-fat meal and a soup made from dried black bean<br>• fiber matched soup was matched for the soluble and insoluble dietary fiber of black beans<br>• antioxidant capacity matched was supplemented with 300 mg grape seed extract	Biochemical measurements	5 hours	The inclusion of black beans with a typical Western-style meal attenuates postprandial insulin and moderately enhances postprandial antioxidant endpoints in adults with MetS.	[137]
Legumes Soy	Iran	Randomized crossover study	42 postmenopausal women with MetS	1) control diet red meat<br>2) diet with soy-nut<br>3) diet with soy-protein 30 g/day	Body weight and biochemical biomarkers	8 weeks	Soy as a replacement for red meat in eating plan had beneficial effects on features of the MetS, soy-nut being more effective than soy-protein. Soy-nut consumption may ↓ insulin resistance and ↑ glycemic control and lipid concentrations in post-menopausal women with the metabolic syndrome.	[138]
	Italy	Randomized controlled trial	28	30 g/day soy protein	Biochemical and immunometric assays	12 weeks	At the end of the treatment period, 50% of subjects on soy food showed a ↓ reduction in the number of MetS features.	[139]
Cereals	Korea	Cross-sectional study	6845<br>No diabete, hypertension or dyslipidemia	Dietary carbohydrate intake assessment (including total carbohydrate, energy from carbohydrates,	FFQ<br>Anthropometric measurements<br>Blood analyses	2007-2009	Percentage of energy from carbohydrates in men.<br>↓ DBP<br>↓ HDL-C level<br>↑ triglyceride levels	[141]

			dietary glycaemic index, dietary glycaemic load, total grains, refined grains, and white rice)			↑ fasting blood glucose levels<br>rice intake in women<br>↑ systolic blood pressure<br>↑ fasting blood glucose<br>↑ triglyceride levels<br>↓ HDL-C level	
Italy and Filland	Randomized control trials	123	1) Diet based on whole-grain cereal products<br>2) Diet based on refined cereal products	Blood plasma analyses	12 weeks	Insulin sensitivity indices and secretion (SI, QUICKI, DI, dAIRG) and lipids and inflammatory markers concentrations (hs-CRP, IL-6, IL-1ra, and TNF-α) no changes after the intervention, and between groups.	[146]
Italy	Randomized controlled intervention study	54	1) Diet based on whole-grain cereal products<br>2)Diet based on refined cereal products	Blood plasma analyses (postprandial glucose, insulin and lipid metabolism)	12 weeks	With respect to the run-in period:<br>↓ 29% postprandial insulin<br>↓ 43% triglyceride responses<br>with respect to group 2:<br>postprandial insulin<br>triglyceride<br>responses were significantly lower.	[147]
China	Cohort study	2892<br>Healthy adults<br>1088 Northern China<br>1804 South China	Quartiles of monthly consumption of rice, wheat and products, coarse, tuber	Semiquantitative FFQ<br>Anthropometric, blood pressure, and biochemical measurements	6 years follow-up	Rice consumption was inversely associated with a risk of MetS<br>wheat and wheat products was positively associated with a risk of MetS.	[148]
Korea	Cohort study	5717<br>without MetS	Grain consumption (whole grains or refined grains) classified:<br>Rare consumers: <1 serving/d,<br>1 to <3 servings/d,<br>Frequent consumers: ≥3 servings/d	Semiquantitative FFQ<br>Anthropometric, blood pressure, and biochemical measurements	10 years<br>follow-up	Frequent consumers of whole grains had a lower risk of incident MetS, of abdominal obesity, high blood glucose, hypertriglyceridemia, and elevated blood pressure.<br>Frequent consumption of refined grains was associated with a higher risk of all 5 components of MetS in women.<br>hypertriglyceridemia, and low HDL-C	[149]
China	Case-control study	667<br>with MetS	whole-grain wheat and rye intake	Plasma DHPPA concentrations<br>Anthropometric measures		Higher plasma DHPPA concentrations were associated with lower risk of MetS.	[150]
	In vivo study	7 week-old male Wistar rats	Cholesterole enriched diet with:<br>1)20% of RP-A<br>2)20& RP-E<br>3)20% CAS	Plasma analyses (amino acid, glucose, albumin, lipids, lipoprotein)<br>Deposit of fat<br>Liver lipid analyses<br>Enzyme activities	2 weeks	Group 1 and 2<br>↓ body weight gain,<br>↓ plasma glucose and lipid levels,<br>↓ hepatic lipids accumulation<br>↓ fatty acid synthase,	[151]

							↓ glucose 6-phosphate dehydrogenase<br>↓ malate dehydrogenase<br>No significant differences were observed between Group 1and 2.	
Olive and fish oil	Brazil	Clinical trial	• fish oil group (n=21)<br>• extra-virgin olive oil group (n=13)<br>• fish oil and extra-virgin olive oil group (n=26)<br>• control group (n=42)	1) 3 g/d of fish oil n-3 fatty acids;<br>2) 10 mL/d of extra-virgin olive oil at lunch and dinner;<br>3) 3 g/d of fish oil n-3 fatty acids and 10 mL/d of extra-virgin olive oil	Biochemical and inflammatory biomarkers measurements, and anthropometric and blood pressure measurements	3 months	Increased dietary n-3 polyunsaturated fatty acids and extra virgin olive oil have beneficial synergistic effects on lipid metabolism and oxidative stress in patients with MetS	[155]
Olive oil	Italy	Clinical trial	25	10 g/day extra virgin olive oil	Biochemical biomarkers	After 2 h meal	After 2 h after meal, subjects who assumed a meal with EVOO had significantly lower blood: ↓ glucose<br>↓ DPP-4 protein and activity<br>↓ LDL-C and ox-LDL<br>↑ higher insulin,<br>↑ GLP-1<br>↑ GIP<br>EVOO improves post-prandial glucose and LDL-C.	[154]
Omega 3	USA	Cross-sectional study	4941	Quintiles of dietary ω-3 fatty acids (g/d)<br>Q1: 0.00-0.07 (n=970)<br>Q2: 0.08-0.14 (n=1070)<br>Q3: 0.15 – 0.21 (n= 918)<br>Q4: 0.22 – 0.37 (n=1009)<br>Q5: 0.38 – 5.65 (n=974)	FFQ	N/A	Dietary ω-3 PUFAs were not associated with prevalent MetS (p for trend = 0.97).	[163]
	Iran	Cross-sectional study	420<br>Female adults	Energy-adjusted fish intake (g/day) Tertiles:<br>Never or less than 1/month<br>1-3 times/month<br>1 time/week<br>2-4 times/week<br>5-6 times/week<br>1-2 times/day	Semiquantitative FFQ	1 year	Highest tertile of fish intake were 65% less likely to have the MetS than those in the lowest tertile high fish intake<br>↓ hypertriglyceridemia ↑ HDL<br>↓ blood pressure	[168]
	Iran	Cohort study	3382<br>adults	Canned and non-canned fish consumption:<br>- < 30 g/week	FFQ	3.6 year follow-up	↓ incidence of MetS<br>in > 45 g/week group	[169]

			- 30-45 g/week<br>- > 45 g/week				
Iran	Cross-sectional study	2451<br>adults	Intake of ALA, EPA and DHA<br>Content of ω3 and ω6 PUFAs	Semiquantitative FFQ<br>Anthropometric measurements<br>blood pressure<br>fasting plasma concentrations of glucose and lipids	followed up every 3 years	Highest quartile of ALA and ω-6 fatty acid intakes had a 38% and a 0.47% lower prevalence of MetS, respectively<br>ω-6/ω-3 PUFA ratio was not associated with a decrease in the prevalence of the MetS.	[170]
USA	Cohort study	4356<br>Young adults free from MetS and diabetes	Fish intake:<br><1/month;<br>1–3/month;<br>1/week;<br>2–4/week;<br>≥5/week.<br>Fish oil<br>(Quintiles, median (g/day)):<br>Q1: 0.03;<br>Q2: 0.07;<br>Q3: 0.11;<br>Q4: 0.18;<br>Q5: 0.33.	FFQ	25 year follow-up	1069 incident cases of MetS<br>ω3-PUFA intake inversely associated with the incidence of MetS in a dose–response manner (hazards ratio of incident MetS was 0.54 (P for linear trend < 0.01) as compared the highest to the lowest quintile of ω3-PUFA intake).	[171]
Korea	Cross-sectional study	1520<br>Prediabetes and normal	Fish consumption:<br>1) ≤ 17.0 g/day<br>2) 18.0-93.0 g/day<br>3) ≥ 94 g/day	FFQ<br>Blood samples	N/A	18.0–93.0 g/day fish consumption<br>↓ total cholesterol 0.422 fold<br>≥ 94 g/day fish consumption<br>↓ total cholesterol 0.555 fold<br>↑LDL-C 2.482 fold	[172]
Norway	Cross-sectional study	23907<br>adults	Fish (fatty, lean and processed) consumption per week:<br>Never<br><1<br>1<br>2–3<br>4–5<br>Approximately every day	FFQ<br>Anthropometric measurements<br>Blood samples	13-year follow-up study	Lean fish consumption once a week or more<br>↓ future metabolic score, ↓ triglycerides<br>↑ HDL-C<br>↓ WC (men)<br>↓ blood pressure<br>Fatty fish consumption<br>↑ WC<br>↑ HDL-C (men)	[173,174]
Australia	*In vivo* study	High-fat induced MetS male Wistar rats	1) corn starch (C; weighing 340±2 g)<br>2) corn starch+ALA-rich chia oil (CA; 339±2 g)<br>3) corn starch+EPA oil (CE; 338±1 g)<br>4) corn starch+DHA oil (CD; 337±2 g)<br>5) high-carbohydrate, high-fat (H; 339±3 g)<br>6) high carbohydrate, high-fat+ALA-rich chia oil (HA; 336±2 g)	Cardiovascular measurements<br>Body composition measurements<br>Fatty acids profile<br>Plasma biochemistry<br>Histology	8 weeks	ALA<br>induced lipid redistribution away from the abdominal area<br>↓ glucose tolerance<br>↓ insulin sensitivity<br>↓ dyslipidemia<br>↓ hypertension and left ventricular dimensions. EPA and DHA	[175]

			7) high-carbohydrate, high-fat+EPA oil (HE; 332±2 g)<br>8) high-carbohydrate, high-fat+DHA oil (HD; 337±1g)			↑ sympathetic activation<br>↓ reduced abdominal adiposity<br>↓ total body fat<br>↓ insulin sensitivity<br>↓ dyslipidemia<br>↓ hypertension and left ventricular stiffness<br>ALA, EPA and DHA<br>↓ inflammation in both the heart and the liver<br>↓ cardiac fibrosis<br>↓ hepatic steatosis<br>↓ stearoyl-CoA desaturase 1 activity	
Brazil	Randomized controlled trial	87 postmenopausal women with MetS	Diet plus 900 mg of ω-3s/day	Anthropometric measurements<br>Blood analyses	24 weeks	↓ SBP(-12.2%) blood pressure<br>↓ DBP (-8.2%)<br>↓ serum triglycerides concentration (-21.4%)<br>↓ insulin resistance (-13.1%) ($P<0.05$)<br>↓ serum IL-6 concentration (-28.5%) ($P=0.034$)	[176]
USA	Randomized, single-blind, parallel intervention study	59<br>with early-stage T2D or MetS	Dietary Supplementation with:<br>1) corn oil (CO)<br>2) botanical oil (BO) combination enriched in alpha-linolenic, gamma-linolenic, and stearidonic acids<br>3) fish oil (FO) enriched in eicosapentaenoic and docosahexaenoic acids	Blood analyses<br>Fatty Acids profile	8 weeks	BO<br>↓ total and LDL-C levels<br>FO<br>↓ serum triglycerides<br>↓ hemoglobin A1c<br>↑ HDL-cholesterol	[177]
Spain	Double-bind randomized intervention	36<br>with MetS	High-intensity interval training and diet:<br>1) 500 mL/day of semi-skim milk (8 g of fat; placebo milk)<br>2) 500 mL/day of skim milk enriched with 275 mg of ω-3PUFA and 7.5 g of oleate (*ω*-3+OLE)	Anthtopometric measurements<br>Cardiometabolic measurements<br>Blood analyses<br>Insulin sensitivity	24 weeks	ω-3+OLE<br>↑ HDL<br>↓ area under the curve of insulin concentration<br>↓ circulating proinflammatory stress	[178]
Spain	Multicenter randomized crossover clinical trials	273<br>with MetS	1) 100 g/d of white fish (Namibia hake) with advice on a healthy diet<br>2) health diet avoiding fish	Lipid profile<br>individual components of the MetS<br>serum insulin concentrations<br>homeostasis model of insulin resistance<br>serum C-reactive protein<br>serum fatty acid levels	8 weeks	↓ WC<br>($P < 0.001$)<br>↓ DBP(P Z 0.014)<br>↓ serum<br>LDL concentrations (P Z 0.048)<br>↑ serum EPA and DHA fatty acids ($P < 0.001$)	[179]

Polyphenols Flavonoids	Spain	Randomized controlled trial	200 with high cardiovascular risk	Participants in both Med-diet intervention groups were given personalized advice about dietary changes<br>1)Med-EVOO participants received free EVOO (1 L/wk)<br>2)Med-nuts group was provided with mixed nuts (30 g/d, as 15 walnuts, 7.5 g almonds, and 7.5 g hazelnuts)	Anthropometrics and clinical parameters were measured as well as BP, plasma NO and TPE in urine samples	4,8 years	Flavonoids increase of endothelial nitric oxide synthase (eNOS) expression and thus nitric oxide production.	[187]
	United Kingdom	Health professionals follow-up study	43880 healthy participants who had no prior diagnosed CVD or cancer	-	FFQ	24 years	Higher intakes of fruit-based flavonoids were associated with a lower risk of nonfatal MI and ischemic stroke in men.	[193]
	Netherlands	Prospective cohort studies	774 elderly Dutch men	• 15.2 mg/day of catechin	Evaluation of risk of CHD mortality	25 years	The risk for death from CVD and CHD decreased by catechin intake.	[194]
	Italy	Randomized controlled trial	90 elderly individuals without clinical evidence of cognitive dysfunction	Consume daily drink containing:<br>• 993 mg [high flavanol (HF)]<br>• 520 mg [intermediate flavanol (IF)]<br>• 48 mg [low flavanol (LF)] cocoa flavanols (CFs)	Parameters evaluated: body and visceral fat weights, and hepatic free fatty acids	8 weeks	This dietary intervention study provides evidence that regular CF consumption can reduce some measures of age-related cognitive dysfunction, possibly through an improvement in insulin sensitivity.	[196]
	Japan	Cohort study	272 without clinical CVD, type 1 diabetes or other severe diseases	-	Questionnaire for lifestyle habits, physical examination and laboratory assessment	From 2002 to 2007	Equol-producers (individuals with the ability to produce equol are termed 'equol-producers') showed a decrease incidence of CAC, but there was no significant association between dietary isoflavones and CAC.	[197]
	America	Prospective cohorts study	124086	All participants self-reported their diet every four years	Validated semiquantita-tive FFQ	From 1986 to 2011	Higher intake of foods rich in flavonols, flavan-3-ols, anthocyanins, and flavonoid polymers may contribute to weight maintenance in adultho-od and may help to refine dietary recommendations for the prevention of obesity and its potential consequences.	[198]
Polyphenols Chlorogenic acid	China	*In vivo* study	40 male Sprague-Dawley rats	Rats were divided in four groups:<br>1) normal diet<br>2) high-fat diet	Body and visceral fat weights, and hepatic free fatty acids	12 weeks	Chlorogenic acid sup-pressed in dose-dependent manner increases in body and visceral fat weights,	[199]

			3) high-fat diet with a low dose of chlorogenic acid (20 mg/kg body weight<br>4) high-fat diet with a high dose of chloro-genic acid (90 mg/kg body weight)			and hepatic free fatty acids induced by high-fat diet.	
Singapore	*In vivo* study	20 Leprdb/db mice and four C57BL/6 mice (control group)	The mice were treated daily with:<br>1) vehicle<br>2) 250 mg/kg BW met-formin by oral gavage<br>3) 50 mg/kg BW chloro-genic acid, daily	G6Pase expression and activity, hepatic steatosis, lipid profiles and skeletal muscle glucose uptake, glucose tolerance, and insulin sensitivity	2 weeks	CGA inhibited hepatic G6Pase expression and activity, attenuated he-patic steatosis, improved lipid profiles and skeletal muscle glucose uptake, which in turn improve fasting glucose levels, glucose tolerance, insulin sensitivity and dyslipidemia.	[200]
China	*In vivo* study	32<br>female C57BL/BKS mice divided in four groups (two control group and two chlorogenic acid group)	• CGA (80 mg/kg BW) once daily	Body fat, fasting plasma glucose, and glycosylated hemoglobin (HbA1c)	12 weeks	Compared to the control group, the percentage of body fat, fasting plasma glucose, and HbA1c significantly decreased ($p < 0.05$) in the chlorogenic acid group.	[202]
China	*In vivo* study	40<br>rats	Rats were divided into four groups<br>1-2) chlorogenic acid: 1 mg/kg BW/day and 10 mg/kg BW/day (together with a diet rich in cholesterol).<br>3) normal diet<br>4) cholesterol-rich diet	Levels of LDL and HDL cholesterol	28 days	High doses of 5-CQA significantly reduced total and LDL-C and increased HDL-C.	[203]
Australia	Randomized, double-blind, placebo-controlled, crossover trial	23<br>healthy subjects	Subjects were treated with 400 mg of 5-CQA dissolved in 200 mL of low nitrate water	Evaluation of endothelial function -<br>blood samples analysis -<br>sanguine pressure measurement	Only time	Decrease in both SBP and DBP (−2.41 and 1.53 mmHg) in subjects treated with chlorogenic acid.	[204]

Curcumin v3o9l	China	*In vitro* study	-	• 0–50 μM curcumin on 24, 48, or 72 h	MTT assay on 3T3-L1 preadipocytes	10 days	• MCE inhibition- PPARγ and C/EBPα expression regulation; • Decrease lipids accumulation.	[210]
	Italy	Randomized and controlled clinical trial	44 patients with obesity	Food supplement for-mulated to be enteric-coated and containing 800 mg/dose daily of *Curcuma longa* extract (95% of curcumin) complexed with sunflower phospholipids (20% phosphatidylserine) and blended with 8 mg/dose/die of piperine from *Piper nigrum* extract	Anthropome-tric measurements and body composition	30 days	• IL-6 and TNF-α levels reduction due to the curcumin; • Piperine addition improve the bioavai-lability and it reduced curcumin urinary excretion.	[211]
	Iran	Phase III randomized double-blind/ parallel group design	117 with MetS	• Curcuminoids (1 g/day) + piperine (10 mg/day)	Blood samples	8 weeks	• Significant reduction in SOD, MDA and CRP inflammation levels; • Improvement in lipid profile, total and LDL-C, HDL-LDL C, and TG; • Increased bioavai-lability of curcuminoids due to piperine.	[212]
	Egypt	*In vivo* study	110 rats (Sprague Dawley) with T2D	1) Control group fed normal diet 2) Group fed HFD 3) Group supplemented with 80 mg/kg/day curcumin 4) Group treated with	Plasma blood test	60 - 75 days	• TNF-α levels attenua-tion, glucose tolerance and improved lipid profile in curcumin treatment; • Curcumin effects comparable to those of rosiglitazone in T2D.	[213]

				1 mg/kg/day rosiglitazone<br>5) Group treated with 80 mg/kg/day curcumin + 1 mg/kg/day rosiglitazone				
	New South Wales	2 × 2 factorial, randomized, double-blinded, placebo-controlled study	64 participants at high risk of developing diabetes	PL) placebo<br>CC) curcumin tablets (180 mg/day)<br>FO) 1.2 g DHA + EPA<br>CC+FO) dual-action tablets of 180 mg curcumin plus 2 capsules providing 1.2 g DHA + EPA	Erythrocyte fatty acid analysis	12 weeks	• Circulating levels of GSK-3β were significantly lower in the curcumin group;<br>• Uncertain benefit on glycaemic control regarding the complementarity of curcumin and omega-3 polyunsaturated fatty. acids	[214]
	Iran	Randomized double-blind clinical trial	50	• 80 mg/day of nanocurcumin	Blood sample and anthropometricmeasurements	12 weeks	• Reduced mean change in triglyceride levels and pancreatic β-cell function;<br>• No significant differences in anthropometric measurements, blood pressure and biochemical factors.	[216]
	France	*In vivo* study	48 mice	• Risperidone<br>• Risperidone + curcumin (0,5 % day)	Inguinal, periovarian adipose tissues and liver collected for biochemical and histological examination	22 weeks	• Reduction of risperidone-induced hepatomegaly by suppression of the ability to induce hepatic overexpression of enzymes involved in lipid metabolism (LXRα, FAS, ACC1, LPL, PPARγ, ACO, SREBP-2);<br>• Decreased glucose intolerance and triglyceridemia;<br>• Decrease in values serum markers of hepatotoxicity and hepatic pro-inflammatory transcription factor NFκB.	[217]
Prebiotics and Probiotics	Iran	Randomized double-blind parallel-group clinical trial	120 adults	6 g/day of probiotics containing freeze-dried *Lactobacillus acidophilus*, *Bifidobacterium bifidum*, *Bifidobacterium lactis* and *Bifidobacter longum* (1.5 × 10^9 each)	Analysis of blood pressure and plasma lipid and lipoprotein concentrations testing	24 week (intervention period) 36 week (follow-up)	Reduction in the prevalence of hyperglycemia in the probiotic and symbiotic groups and a decrease in the tendency to develop hypertension in the probiotic group.	[218]

Spain	Randomized, double-blind crossover placebo-controlled, single-center trial	53	*L. reuteri* V3401 (5×10^9 colony-forming units) + Healthy lifestyle	Biochemical characteristics and inflammatory biomarkers in blood samples	12 weeks	IL-6 and sVCAM effects, but no differences in the clinical features.	[219]

EVOO: extra virgin olive oil; BP: Blood pressure; TPE: total polyphenol excretion; FFQ: food frequency questionnaires; eNOS: endothelial nitric oxide synthase; CVD: cardiovascular disease; MI: Myocardial Infarction; NO: Nitric Oxide; TPE: total polyphenol excretion; CHD: Coronary heart disease; HF: High flavanol; IF: Intermediate flavanol; LF: Low flavanol; CFs: Cocoa flavanols; BW: Body weight; BMI: Body mass index; CAC: coronary artery calcification; CGA: Chlorogenic acids; G6Pase: Glucose-6-phosphatase; HbA1c: glycosylated hemoglobin; LDL: Low-density lipoprotein; LDL-C: Low-density lipoprotein cholesterol; HDL: High-density lipoprotein; HDL-C: High-density lipoprotein cholesterol; 5-CQA: 5-caffeoylquinic acid; SBP: systolic blood pressure; DBP: diastolic blood pressure; MCE: mitotic clonal expansion; PPARγ: Peroxisome Proliferator-Activated Receptor Gamma; C/EBPα: CCAAT-enhancer binding protein α; IL-6: Interleukin 6; TNFα: Tumor necrosis factor alpha; MDA: Malondialdehyde; CRP: C-Reactive Protein; TG: triglyceride; HFD: High-fat diet; GSK-3β: Glycogen synthase kinase-3 beta; ALA: Alpha linolenic acid; DHA: Docosahexaenoic acid; EPA: Eicosapentaenoic acid; ω-3PUFAs: ω3-polyunsaturated fatty acids; LXRα: Liver X receptor alpha; FAS: Fas cell surface death receptor; ACC1: Acetyl-CoA carboxylase; LPL: Lipoprotein lipase; ACO: Acyl CoA Oxidase; SREBP-2: Sterol regulatory element-binding transcription factor 1; TAC: Total antioxidant capacity; FLI: fatty liver index; ALE: Artichoke (*Cynara scolymus*) leaf extracts; FBS: Fetal bovine serum; GPx: Glutathioneperoxidase; SOD: superoxide dismutase; RBC: Red blood cells; HFHC: High fat and High carbohydrates ; AMPKa: AMP-activated protein Kinase; T2D: Type 2 diabetes; PGC-1α: Peroxisome proliferator-activated receptor-gamma coactivator; WT: Wild type; DHPPA: 3,4 dihydroxyphenylpropionic acid; DPP-4: Dipeptidyl peptidase-4; GLP-1: Glucagon-like peptide 1; GIP: Gastric inhibitory polypeptide; sVCAM: Soluble Vascular Cell Adhesion Molecule-1; WC: Waist circumference
